# Microalgae-Based Biostimulants Improve Biomass Production and Root-Linked Performance Stability in *Pelargonium*: A Three-Year Greenhouse Study

**DOI:** 10.3390/plants15050803

**Published:** 2026-03-05

**Authors:** Attila Németh, Wogene Kabato, Nándor Horváth, Norbert Fehér, Zoltán Molnár

**Affiliations:** 1 Albert Kázmér Faculty of Agricultural and Food Sciences, Széchenyi István University, 9026 Gyor, Hungary; kabato.wogene.solomon@ga.sze.hu (W.K.); horvathnandor1989@gmail.com (N.H.); 2 Department of Logistics and Management Informatics, Faculty of Business Administration Zalaegerszeg (GKZ), University of Pannonia, 8200 Zalaegerszeg, Hungary; feher.norbert@zek.uni-pannon.hu

**Keywords:** microalgal biostimulants, protected cultivation, ornamental horticulture, *Pelargonium* spp., biomass accumulation, root architecture, sustainable greenhouse production

## Abstract

Microalgae-based biostimulants may help reduce inputs in protected ornamental production, yet evidence across multiple growing seasons remains limited. We conducted a three-year polytunnel trial with six *Pelargonium* cultivars and applied two strains from the Mosonmagyaróvár Algal Culture Collection (MACC-612, *Nostoc piscinale*; MACC-922, *Chlorella vulgaris*). Using a factorial general linear model, we detected significant treatment effects on total dry mass, root mass, plant height, and root-collar diameter (all *p* < 0.001). Relative to the control, dry mass increased by 19.2% with MACC-612 and 33.1% with MACC-922, while root mass increased by >22% under both treatments. Root-collar diameter was strongly associated with overall plant size (R^2^ = 0.89). Treatment × year interactions were not significant (*p* > 0.05), providing no statistical evidence of season-specific treatment effects within the three-year trial. Cultivars differed mainly in response magnitude rather than direction. Overall, microalgae applications improved biomass accumulation and root-linked structural traits associated with plant vigor under this production system.

## 1. Introduction

Sustainable horticulture is increasingly expected to achieve high yields and consistent plant quality while simultaneously lowering environmental burdens and improving the efficiency of resource use. In greenhouse ornamental production, growers frequently rely on intensive fertilization regimes and chemical growth regulators to obtain compact plant architecture and uniform biomass formation, practices that are associated with growing environmental and regulatory concerns [1]. As a result, the identification and implementation of innovative, environmentally sound cultivation strategies has become a priority in sustainable agriculture and protected cropping systems [2].

Plant biostimulants are used to support growth by influencing a plant’s own physiological and metabolic processes, rather than by supplying nutrients directly [3]. Reported benefits include better nutrient uptake, improved tolerance to abiotic stresses and more uniform growth under variable conditions [4]. In greenhouse and other controlled systems, these products are often explored as a way to maintain quality while reducing fertilizer or other inputs [5].

Among the different categories of biostimulants, products derived from microalgae represent a particularly dynamic and expanding area of research. Microalgae are rich in bioactive constituents—including phytohormones, amino acids, polysaccharides, and essential micronutrients—that are capable of influencing plant metabolic pathways and growth regulation [6]. Beyond their direct physiological effects, microalgae cultivation systems can be linked to wastewater utilization and carbon sequestration processes, thereby supporting circular bioeconomy concepts and contributing to more resource-efficient agricultural production [7]. Experimental studies have shown that microalgae-based preparations can modify root architecture and improve nutrient assimilation, ultimately promoting structural biomass formation [8]. At the same time, both regulatory developments and functional research increasingly emphasize the need for scientific validation and standardized application frameworks for biostimulants in modern horticulture [9]. Accordingly, we tested two MACC strains—MACC-612 (*Nostoc piscinale*) and MACC-922 (*Chlorella vulgaris*)—as complementary biostimulant source types (cyanobacteria vs. green microalgae); a brief selection rationale and examples of commonly investigated genera. 

Recent research underlines the importance of evaluating biostimulant efficacy over multiple growing seasons in order to assess the consistency and reliability of plant responses under practical production conditions [10]. In controlled-environment horticulture, production consistency and plant handling quality have emerged as important topics [11]. Evidence from greenhouse trials indicates that biostimulant treatments can positively influence vegetative growth and ornamental quality parameters [12]. In this context, alterations in root system architecture are widely regarded as a key mechanism driving improved nutrient uptake efficiency and overall plant performance [13]. Microalgae extracts have been reported to stimulate root growth and nutrient acquisition in various horticultural species [14], and broader evaluations confirm their capacity to enhance tolerance to abiotic stress factors [15].

In ornamental species such as *Pelargonium*, structural characteristics—including root biomass and root collar diameter—play a decisive role in mechanical stability, transplant success, and marketable quality. Nevertheless, information on the year-to-year stability of microalgae-induced growth responses and on genotype-specific variability in structural traits remains scarce under greenhouse substrate-based production systems. Accordingly, the present study investigates the impact of two microalgae-based treatments on biomass accumulation and structural growth parameters in six *Pelargonium* cultivars over three consecutive production seasons. We hypothesized that microalgae application would promote structural biomass development and root system enhancement, that these responses would be directionally consistent across seasons under the tested conditions, and that cultivar-related differences would be expressed primarily in response magnitude while maintaining an overall positive trend.

The aims of this study were to: (i) quantify the effects of two microalgae-based biostimulant treatments on above- and belowground biomass production and key quality traits in greenhouse-grown *Pelargonium*; (ii) evaluate whether the biostimulant responses were consistent across cultivars and growing seasons (three-year study), including the assessment of treatment × cultivar and treatment × year interactions; and (iii) assess the relationship between root-linked traits and overall growth stability under variable seasonal conditions.

## 2. Results

The general linear model showed significant treatment effects on all measured biomass and structural traits (Table 1). Treatment significantly affected total dry mass, root mass, root-collar diameter, and plant height (*p* < 0.001 for each). Treatment × Year interactions were not significant for any parameter (*p* > 0.05), providing no statistical evidence that treatment effects differed among the three growing seasons. The trial included three regimes: control, MACC-612, and MACC-922 (Table 2).

While the study was not designed as an equivalence test across years, the maintained direction of response and consistent treatment ranking across the three seasons support an operationally relevant consistency of the observed benefits under the tested greenhouse conditions. Significant Treatment × Cultivar effects (Table 1) indicate that cultivars mainly differed in response magnitude. Given *n* = 3 per cultivar × treatment × year cell, the non-significant Treatment × Year term is interpreted as no evidence of an interaction under the tested conditions rather than proof of stability. A compact year-wise summary (Δ% vs. control; averaged across cultivars) is provided in Supplementary Materials S9 and shows directionally consistent increases across seasons—for example, dry mass increased by +18.5% to +27.5% (T1) and +33.4% to +35.0% (T2), while root mass increased by +16.6% to +31.0% (T1) and +22.6% to +29.5% (T2).

In total, six *Pelargonium* cultivars (Table 3) were tested under three treatment regimes, including two microalgae-based applications in addition to the control.

During plant selection, priority was given to widely marketed ornamental cultivars in order to ensure practical applicability and facilitate repeatability of the trials in subsequent production seasons. The impact of the microalgae-based treatments was assessed across three commercially significant *Pelargonium* groups: *Pelargonium* × *Zonale*, *Pelargonium* × *Peltatum*, and *Pelargonium* × *Lateripes*. The specific cultivars included in the study are presented in Table 3. In this study, we tested two MACC strains—MACC-612 (*Nostoc piscinale*) and MACC-922 (*Chlorella vulgaris*)—to represent complementary biostimulant source types (cyanobacteria vs. green microalgae) under identical greenhouse conditions. More broadly, commonly investigated genera in plant biostimulant research include *Chlorella*, *Arthrospira* (*Spirulina*), *Scenedesmus*, *Dunaliella*, *Nannochloropsis*, and cyanobacteria such as *Nostoc* and *Anabaena*. Detailed selection rationale is provided in Section 4.2. Accordingly, we selected *Chlorella vulgaris* as the green microalga due to its applied horticultural relevance and established use in biomass-based biostimulant testing, whereas microalgae primarily used as laboratory models (e.g., *Chlamydomonas reinhardtii*) were not the focus of this greenhouse performance study.

### 2.1. Dry Mass Responses

**Figure 1:**
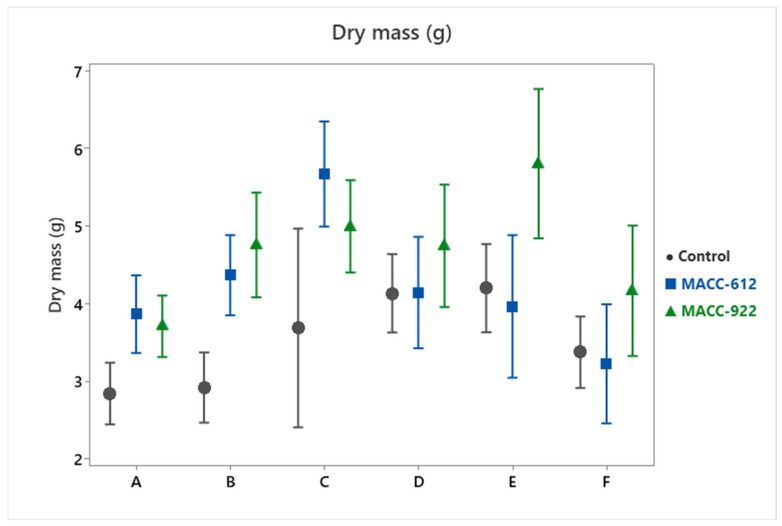
Dry mass (g) of six *Pelargonium* cultivars under control and microalgae treatments (MACC-612 and MACC-922). Points represent means and error bars indicate 95% confidence intervals. Cultivar codes (A–F) are described in Table 3.

Dry mass was substantially increased in response to both microalgae treatments (Table 1; Figure 1). When averaged across cultivars and growing seasons, control plants produced a mean dry mass of 3.53 ± 0.14 g. Treatment with MACC-612 raised this value to 4.21 ± 0.16 g, corresponding to a 19.2% increase, whereas MACC-922 resulted in an average dry mass of 4.7 ± 0.15 g, representing a 33.1% improvement over the control.

Although the Treatment × Cultivar interaction was significant (*p* < 0.001), the magnitude of the response to microalgal applications differed among genotypes, with MACC-922 eliciting a larger increase in some cultivars while both treatments performed similarly in others. Overall, treatment effects were consistently non-negative across cultivars, and the direction of the response was maintained across years, supporting the consistency of the observed pattern.

### 2.2. Root Biomass Enhancement

**Figure 2:**
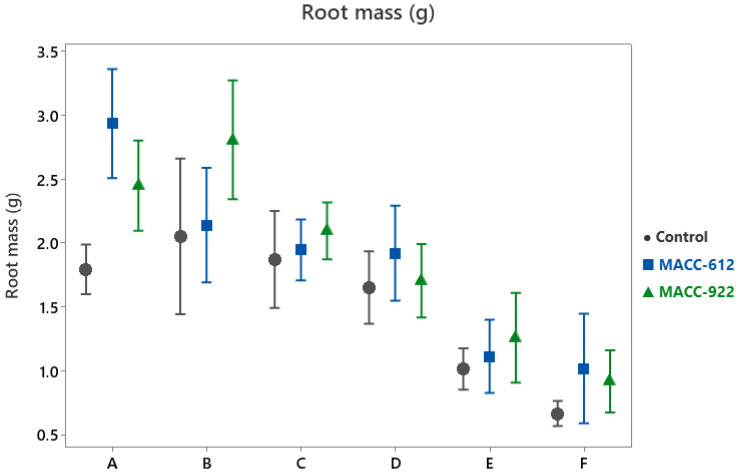
Root mass (g) of six *Pelargonium* cultivars under control and microalgae treatments (MACC-612 and MACC-922) across three consecutive growing seasons (2023–2025). Points represent mean values and error bars indicate 95% confidence intervals. Cultivar codes (A–F) are defined in Table 3.

Root biomass responded markedly to microalgae application (Table 1; Figure 2). In the control treatment, plants developed a mean root mass of 1.51 ± 0.09 g. This value increased to 1.84 ± 0.11 g (+22.3%) with MACC-612 and to 1.87 ± 0.11 g (+24.1%) with MACC-922.

The magnitude of the response varied significantly among cultivars (Treatment × Cultivar, *p* < 0.001). Some genotypes showed particularly pronounced root enhancement under MACC-922, whereas others exhibited moderate yet consistent increases under both treatments. Despite these differences in response intensity, all cultivars demonstrated higher root mass compared with the control.

In most cases, the relative increase in root biomass exceeded that recorded for shoot dry mass, suggesting a shift in biomass allocation toward below-ground organs following microalgae treatment.

### 2.3. Root Collar Diameter Response to Microalgae Treatments

**Figure 3:**
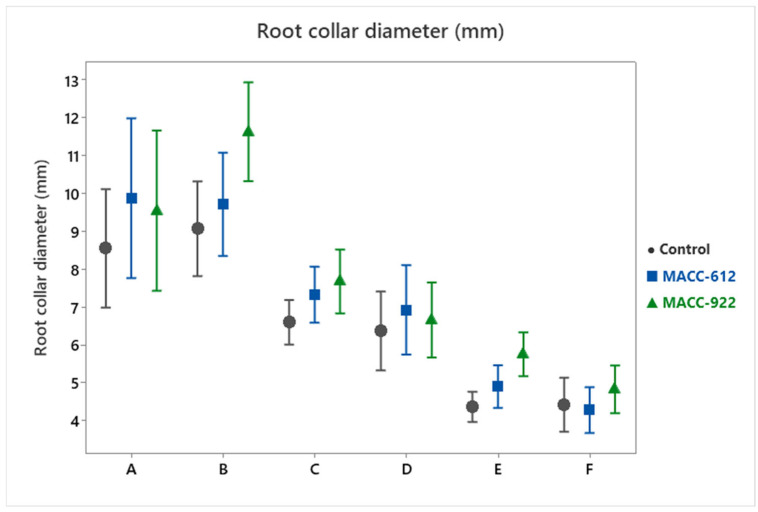
Root collar diameter (mm) of six *Pelargonium* cultivars under control and microalgae treatments (MACC-612 and MACC-922) across three consecutive growing seasons (2023–2025). Points represent mean values and error bars indicate 95% confidence intervals. Cultivar codes (A–F) are defined in Table 3.

Treatment had a significant effect on root collar diameter (Table 1). In the control group, plants developed a mean collar diameter of 6.56 ± 0.30 mm. Application of MACC-612 increased this value to 7.17 ± 0.36 mm (+9.3%), while MACC-922 resulted in a further rise to 7.68 ± 0.37 mm (+17.0%) (Figure 3).

The multi-factor GLM showed high overall fit (R^2^ = 0.8925), which reflects the explanatory power of the full model (all terms combined) rather than the treatment effect alone. The significant Treatment × Cultivar interaction (*p* = 0.006) suggests that the degree of collar thickening differed among genotypes, with some cultivars responding more strongly, particularly under MACC-922. Nevertheless, collar diameter increased relative to the control across cultivars, supporting a broadly consistent positive structural effect.

### 2.4. Plant Height and Growth Balance

**Figure 4:**
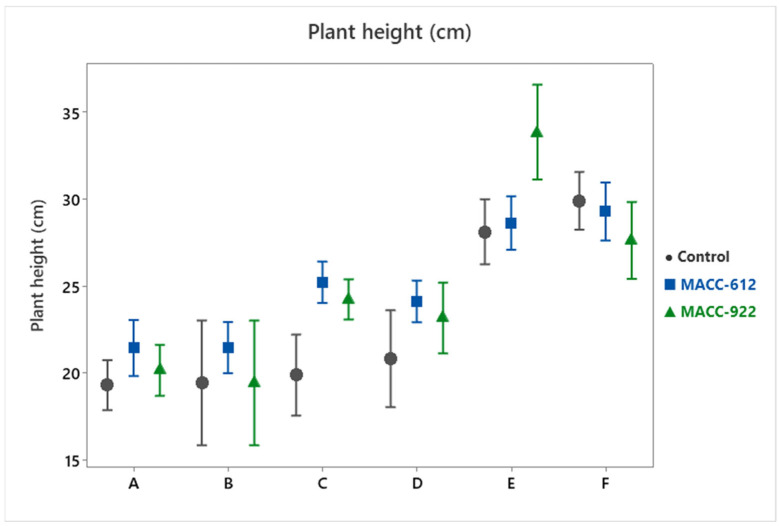
Plant height (cm) of six *Pelargonium* cultivars under control and microalgae treatments (MACC-612 and MACC-922) across three consecutive growing seasons (2023–2025). Points represent mean values and error bars indicate 95% confidence intervals. Cultivar codes (A–F) are defined in Table 3.

Plant height was also significantly influenced by treatment (Table 1; Figure 4), although the relative increases were less pronounced than those observed for biomass-related traits. Control plants attained a mean height of 22.92 ± 0.72 cm. In comparison, plants treated with MACC-612 reached 25.02 ± 0.49 cm (+9.2%), while MACC-922 resulted in a mean height of 24.74 ± 0.78 cm (+8.0%).

In relative terms, the increase in height was smaller than the gains measured for dry mass and root biomass.

This cross-trait comparison is reported descriptively based on the separate trait-wise GLM results and the cultivar-stratified interval/interaction plots, and we do not infer biomass-allocation shifts from percent changes alone.

Formal allocation analyses (e.g., shoot:root ratios) would require an explicit ratio-based modelling approach and are proposed as future work.

### 2.5. Pooled Treatment Response Summary

To make the overall magnitude of the treatment responses more tangible, we summarized pooled treatment means across all cultivars and seasons (n = 54 per treatment) and calculated absolute and relative changes compared with the untreated control (see Supplementary Materials S6). In practical terms, MACC-612 (*Nostoc piscinale*) increased mean dry plant mass from 3.53 g (control) to 4.21 g (+0.68 g; +19.2%), while MACC-922 (*Chlorella vulgaris*) raised it to 4.70 g (+1.17 g; +33.1%). Root mass showed a similarly consistent improvement, increasing from 1.51 g in the control to 1.84 g under MACC-612 (+0.34 g; +22.3%) and 1.87 g under MACC-922 (+0.36 g; +24.1%). Structural traits also improved: root collar diameter increased by +9.3% with MACC-612 and +17.0% with MACC-922, while plant height increased more moderately (+9.2% and +8.0%, respectively). Together, these pooled summaries indicate that both strains primarily promoted biomass accumulation and root-linked structural development rather than causing disproportionate shoot elongation, while the detailed cultivar-level variation is documented separately in Supplementary Materials S8.

### 2.6. Model Diagnostics and Statistical Validation

Model assumptions were evaluated using residual normal probability plots and variance homogeneity tests (see Supplementary Materials S1–S4). Normal probability plots did not indicate major departures from normality. Levene’s test supported homogeneity of variances for dry mass (*p* = 0.494), root mass (*p* = 0.344), and root collar diameter (*p* = 0.414), whereas plant height showed mild heteroscedasticity (*p* = 0.031). Given the balanced factorial design and the limited magnitude of variance differences, the height model was interpreted with emphasis on effect direction and consistency across the dataset. Overall, these diagnostics indicate that the factorial GLM provided an adequate basis for inference.

The significant Treatment × Cultivar interactions detected for dry mass, root mass, and root collar diameter indicate that responsiveness to microalgae application was genotype-dependent rather than uniform across cultivars. Consistency across years was further supported by the absence of significant Treatment × Year interactions for all evaluated *Pelargonium* traits, providing no statistical evidence that treatment ranking differed across the three consecutive growing seasons. Cultivar × Year effects were also examined and did not change the interpretation of the treatment effects. Statistical significance was determined at α = 0.05.

## 3. Discussion

The three-year greenhouse trial offers statistically and biologically well-supported evidence that microalgae-based applications enhanced structural biomass formation and root development in *Pelargonium* grown under substrate-restricted ornamental production conditions. The consistent treatment responses across principal growth variables indicate coordinated modulation of plant development rather than isolated stimulation of a single trait. By incorporating Treatment, Cultivar, and Year into a factorial analytical framework, the study accounted for both genetic variability and seasonal influences, strengthening confidence in the robustness of the observed patterns under the tested conditions.

In this context, we use the term root-linked response consistency to describe treatment responses that remained directionally similar across seasons and were accompanied by root-linked morphological enhancement (increased stem/root growth). Because replication per cultivar × treatment × year cell was n = 3, interaction tests may have limited power to detect small year-specific deviations; accordingly, we emphasize effect direction, treatment ranking, and confidence intervals in interpreting year-to-year consistency. The absence of significant Treatment × Year interactions indicates that we found no statistical evidence that treatment effects differed detectably among the three seasons. Combined with the consistently positive direction of responses across cultivars and years, this supports repeatable benefits within the environmental range covered by the present trial, without implying categorical stability beyond it.

The proportional increase in root biomass relative to shoot elongation suggests preferential below-ground allocation under microalgal treatment. In containerized ornamental systems, where rooting volume is inherently restricted, enhanced root development may improve nutrient interception efficiency and buffering capacity under fluctuating substrate moisture conditions [16]. Such biomass partitioning patterns are frequently associated with improved physiological stability in controlled horticultural production systems.

To place the magnitude of our responses in context, recent greenhouse and horticultural studies on microalgae/cyanobacteria-derived biostimulants report a broad range of biomass and root-related effect sizes that depend strongly on formulation (whole biomass vs. extract), dose and application frequency, and the developmental stage at treatment [17,18]. In particular, repeated foliar/irrigation programs or enriched extracts can yield larger but more variable responses, whereas single, transplant-time applications tend to show more moderate yet operationally attractive effects [17,18]. Against this background, the present results—showing clear increases in biomass and root-linked traits with cultivar-dependent magnitudes—support the view that response size is not a fixed ‘microalgae effect’ but a function of protocol and genotype. Therefore, differences among studies should be interpreted in light of methodological factors (strain cultivation and harvesting, extraction/processing, concentration standardization, and application regime) that directly shape the bioactive profile and, consequently, the expected effect size [17,18,19]. Accordingly, we interpret our effect sizes as practically relevant under a single-application, transplant-time protocol, while recognizing that higher magnitudes reported elsewhere often reflect repeated applications, enriched extracts, or different standardization approaches.

These findings are also consistent with the rapidly expanding literature on microalgae- and cyanobacteria-derived biostimulants across production contexts. For example, microalgal hydrolysates (including *Chlorella vulgaris*) have been reported to promote growth in substrate-grown *Pelargonium*, providing a closely aligned external reference point for our system [13]. Related studies also report biostimulant responses from cyanobacterial or algae-associated products in other production contexts, indicating potential applicability beyond greenhouse ornamentals, depending on formulation and application regime [20,21].

At the mechanistic level, microalgae-derived preparations contain bioactive compounds capable of modulating hormonal signaling pathways and stress-related responses [4,6,22]. Evidence indicates that algal extracts can influence auxin- and cytokinin-mediated developmental processes affecting root architecture and biomass distribution [23,24]. The pronounced root and collar responses observed here are consistent with these mechanisms and support the interpretation of coordinated structural development rather than disproportionate shoot stimulation. Increased collar diameter is particularly relevant in ornamental crops, as basal stem thickness contributes to vascular functionality, assimilate transport efficiency, and mechanical stability during handling and post-production phases [25,26]. Notably, beyond inducing auxin-like responses, some microalgae have been reported to produce indole-3-acetic acid (IAA). Direct evidence for IAA production has been described for the model microalga *Chlamydomonas reinhardtii* under defined conditions [27]. Because phytohormones were not quantified in the applied preparations, we refer to IAA as a plausible contributing mechanism rather than a confirmed driver of the observed root- and growth-related effects.

Genotype-dependent responsiveness aligns with findings from other horticultural systems, where differential sensitivity to biostimulant-derived signals is attributed to variation in endogenous hormonal balance and metabolic baseline activity [28,29]. Importantly, no cultivar exhibited negative deviation from control performance, indicating broad compatibility of the tested strains across distinct genetic backgrounds.

From a practical perspective, multi-season directional consistency is of particular relevance. Multi-season consistency remains insufficiently documented in ornamental biostimulant research, yet it represents a critical prerequisite for commercial implementation [30]. The present findings suggest that microalgae-based growth modulation is not purely transient but is consistent with integration into developmental regulation under greenhouse conditions.

From a sustainability perspective, microalgae-derived inputs may contribute to more resource-efficient horticultural systems by supporting circular bioeconomy concepts, including nutrient valorization and carbon utilization strategies [7,31]. Enhanced root systems and balanced structural development may further support improved resource-use efficiency and buffering capacity against abiotic stress [11,32]. Recent studies in greenhouse horticulture increasingly emphasize biologically derived growth modulators as complementary tools within integrated nutrient management frameworks [33,34]. Advances in microalgae cultivation technologies are also improving the feasibility of scalable biomass production tailored to agricultural applications [31]. While the present study did not quantify reductions in external fertilizer or growth regulator inputs, the documented structural and biomass responses provide a physiological basis for future optimization. Reviews further suggest that microalgae- and cyanobacteria-derived biostimulants can support sustainability goals by combining growth modulation with nutrient recycling and reduced reliance on conventional agrochemical inputs, while noting that cost-effective scale-up and formulation remain key adoption barriers [35].

Recent literature (last 2–3 years) increasingly frames microalgal biostimulant action as multi-component rather than single-factor: soluble metabolites and hormone-related compounds, peptides/amino acids, polysaccharides and antioxidant molecules may contribute concurrently, and indirect effects mediated through rhizosphere microbial communities are frequently emphasized [36,37]. These reports also stress that composition depends on upstream production conditions and downstream processing, which can explain why nominally similar ‘microalgal’ treatments differ in effect size across experiments [36,37]. In this context, our findings are consistent with a coordinated root–shoot developmental response rather than disproportionate shoot stimulation, and the genotype dependence observed here aligns with evidence that plant baseline physiology and hormone homeostasis modulate responsiveness to biostimulant-derived signals.

Beyond their direct biostimulant effects in horticulture, microalgae can be positioned within a circular-bioeconomy logic where environmental services and product valorization are coupled. In ‘waste-to-resource’ schemes, cultivation can be integrated with nutrient and carbon capture (e.g., polishing nutrient-rich streams and converting recovered N/P and CO_2_ into biomass), while downstream biorefinery routes valorize biomass into multiple product streams, including agricultural inputs. In this context, *Chlamydomonas reinhardtii* is relevant not because it was the production strain in our trial, but because it is a model system used to develop and optimize bioremediation-linked cultivation and bioproduct formation. This framing connects to greenhouse biostimulant systems by clarifying how scalable, standardized biomass supply can be achieved and how co-products may improve the economic feasibility of deploying algal inputs in protected horticulture; greenhouse trials such as the present study then evaluate downstream agronomic performance outcomes [38,39].

At the same time, recent evidence shows that rapid screening assays for algal extracts can be confounded by strong seasonal (circannual) biological variation even under controlled conditions, underscoring the need for standardized, multi-temporal validation protocols when selecting strains and extraction methods [40]. The raw end-point measurement dataset used for analysis is provided in the Supplementary Materials S7.

## 4. Materials and Methods

### 4.1. Experimental Site and Design

The trial was carried out over three consecutive growing seasons (2023–2025) in a plastic-covered greenhouse (polytunnel) operated under standard ornamental production practices. Transplanting was performed each year on the Friday of calendar week 8 in accordance with standard commercial scheduling. Plants were harvested 42 days after transplanting, immediately prior to the commercial sale stage, ensuring that all measurements reflected market-ready material. The resulting fixed production windows were 24 February 2023 to 7 April 2023, 23 February 2024 to 5 April 2024, and 21 February 2025 to 4 April 2025. All environmental summaries reported for each season refer strictly to these 42-day cultivation periods. The total cultivation area was approximately 300 m^2^ and was located in Bősárkány, Győr-Moson-Sopron County, Hungary (47.690455° N, 17.251585° E).

Plants were grown in Hawita™ Uni 20-II (Hawita Group GmbH, Vechta, Germany) peat substrate, pre-moistened with tap water prior to pot filling. A slow-release fertilizer (Osmocote™ Exact High K) (ICL Group, Tel Aviv, Israel) was incorporated into the substrate at a rate of 3 g L^−1^ and thoroughly mixed before potting.

Irrigation was applied daily throughout the cultivation period. In addition to the incorporated slow-release fertilizer, nutrient supply was adjusted via a Dosatron proportional injector system. During the first two weeks after transplanting, a phosphorus-dominant nutrient solution was applied to support early root establishment. This was followed by a nitrogen-dominant formulation for weeks 3–4 to promote vegetative development. From week 5 until harvest, a potassium-dominant formulation was used to enhance structural stability and overall plant quality.

The nutrient solution pH was maintained at 5.8. Electrical conductivity (EC) was set initially at 1.5 mS cm^−1^ and gradually increased to 2.0 mS cm^−1^ as plant demand increased. The EC of the source water was 0.3 mS cm^−1^. To stabilize solution pH at 5.8, 0.2 L of 65% nitric acid was added per 2000 L of irrigation water. Nutrient solution pH and EC were monitored using an Agrár 2000 pH meter(Pronova Analysentechnik GmbH & Co. KG, Berlin, Germany) and a GroLine HI98331 EC meter (Hanna Instruments, Woonsocket, RI, USA), respectively.

**Figure 5:**
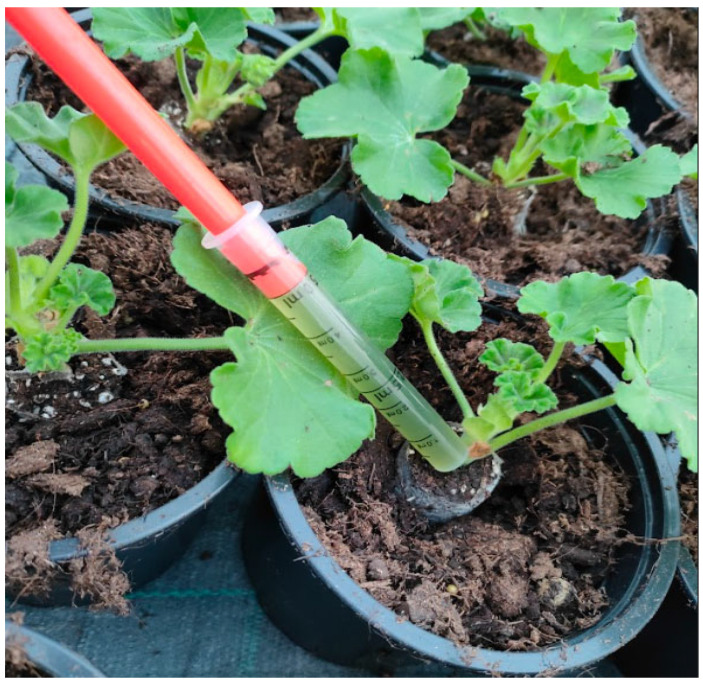
Application method of microalgae suspension. A 1 mL dose (1 g/L) was applied directly to the root collar zone of each transplanted cutting using a precision syringe to ensure uniform treatment delivery, one time on the first day after transplantation.

Uniform, commercially rooted vegetative cuttings representing six *Pelargonium* cultivars were transplanted into Teku VCG pots (⌀120 mm; 0.69 L) containing a peat-based growing medium. The microalgae treatments were administered at the time of transplanting (Figure 5).

A fully randomized factorial design was applied:

• Treatment (3 levels: Control (K), MACC-612 (T1), MACC-922 (T2));
• Cultivar (6 levels: A–F);
• Year (3 levels: 2023, 2024, 2025);
• For each cultivar × treatment combination, 3 biological replicates were cultivated per season.

The experimental unit was an individual potted plant. In each growing season, three independent biological replicates were established per cultivar × treatment combination (n = 3).

Because two cultivars represented each growth habit category (upright, semi-trailing, trailing), the per-season sample size was six plants per growth habit × treatment combination (2 cultivars × 3 replicates). Across six cultivars and three treatments, a total of 54 plants were evaluated per season (6 cultivars × 3 treatments × 3 replicates), corresponding to 162 plants over the three-year experimental period.

All statistical analyses were conducted at the individual plant level using a factorial General Linear Model including Cultivar as an independent factor.

At the start of each growing season, pots were spatially interspersed to minimize positional bias within the polytunnel; plant positions were then kept fixed throughout the production cycle to avoid introducing additional variability from handling and repositioning.

Temperature and relative humidity were checked operationally to avoid extreme conditions; however, RH was not continuously logged and no written records were retained.

Within each season, plants were arranged in a completely randomized design within the polytunnel. Cultivar and treatment combinations were spatially interspersed to minimize positional bias related to edge effects or environmental gradients. Plant positions were reassigned between seasons, and no systematic block structure was imposed. All treatments were distributed evenly across the available cultivation area.

### 4.2. Species Selection Within the Microalgal Biostimulant Context

Microalgae-based biostimulants have gained increasing attention in greenhouse horticulture due to their capacity to modulate plant growth through bioactive metabolites rather than direct nutrient supply. Recent comprehensive reviews emphasize that *Chlorella vulgaris* represents one of the most consistently investigated taxa in applied crop systems, owing to its reliable cultivation characteristics and reproducible physiological effects [11,41]. In horticultural contexts, *Chlorella*-derived preparations have demonstrated measurable improvements in biomass accumulation and root development under controlled production systems [13,14].

In contrast, while *Chlamydomonas reinhardtii* has been extensively used in physiological and molecular plant research, its application in commercial crop production remains limited [14]. The selection of *Chlorella vulgaris* and *Nostoc piscinale* in the present study was therefore guided by their demonstrated agronomic feasibility and previously documented growth-modulating effects in horticultural environments [11,13].

### 4.3. Cultivation, Harvesting, Biomass Processing, and Dose Standardization of MACC Microalgal Strains

**Figure 6:**
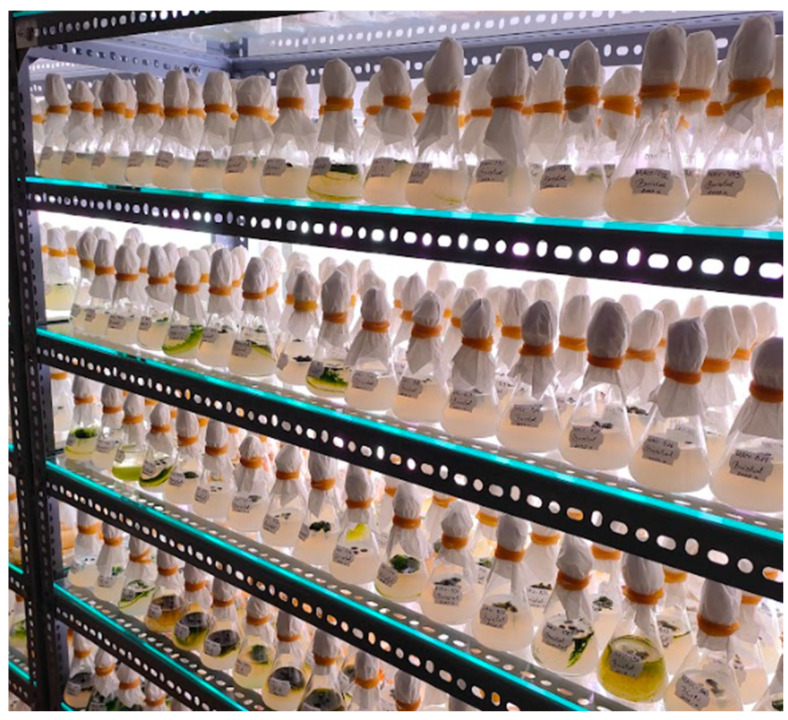
Example of the MACC laboratory cultivation setup (culture vessels on illuminated shelves) used to maintain and grow the investigated microalgal strains prior to harvest.

The microalgal strains MACC-612 (*Nostoc piscinale*) and MACC-922 (*Chlorella vulgaris*) were obtained from the Mosonmagyaróvár Algal Culture Collection (MACC, Mosonmagyaróvá, Hungary) and maintained/cultivated according to the collection’s routine protocol. Stock cultures were kept on solid medium at 15 ± 2 °C under dim light, and experimental cultures were initiated in liquid media selected by taxonomic group (Cyanobacteria: Zehnder-8 or BG-11; Chlorophyta/Charophyta: Tamiya or Bristol). Cultivation (Figure 6) was performed under controlled conditions (25 ± 2 °C; 12:12 h light/dark; ~130 µmol photons·m^−2^·s^−1^; continuous aeration with sterile air and 1.5% CO_2_ enrichment during the light period), starting from 10 mg·L^−1^ dry weight (DW). Cultures were harvested at the stationary growth phase (typically 8–21 days, depending on strain).

Biomass was collected by centrifugation (2150× *g*, 15 min, room temperature), freeze-dried, and stored at −18 °C until use. For treatments, freeze-dried biomass was resuspended in distilled water, thoroughly mixed, and sonicated for 3 min; the working dose was standardized on a DW basis (1 g·L^−1^ = 1.0 g DW per 1.0 L final volume), ensuring comparable concentrations across strains. The selected dose (1 g·L^−1^) was chosen as the highest level of a logarithmic dose range commonly used in preliminary screening to maximize detection of strain-dependent bioactivity while avoiding non-physically relevant application levels. At transplanting, 1 mL of the suspension was applied directly to the root-collar zone of each plant using a single-use plastic syringe (1 mL) as a single application; control plants received 1 mL of distilled water. Fresh treatment suspensions were prepared separately for each growing season following the same protocol, and biomass amounts were determined gravimetrically to maintain consistent application rates across years. Strains were cultivated under controlled laboratory conditions and handled aseptically throughout harvesting, processing, and transport; however, axenicity (bacteria-free status) was not formally verified by microbiological testing in this study.

### 4.4. Hormone-like Compounds and Root Development

The growth-promoting effects attributed to microalgae-based treatments are frequently associated with the presence of hormone-like compounds and signaling metabolites. Early experimental evidence confirmed cytokinin- and auxin-like activity in microalgae and cyanobacteria [42], while more recent analyses describe the capacity of microalgal extracts to influence root system architecture and biomass allocation patterns [13,30]. The observed stimulation of root biomass in the present study is therefore consistent with previously described interactions between algal-derived metabolites and endogenous plant hormonal regulation pathways.

Although direct phytohormone quantification was not performed in this experiment, the coordinated increase in below-ground biomass aligns with established models of auxin-mediated modulation of root elongation and lateral root formation in plant stress physiology [28,29].

### 4.5. Microalgae-Based Inputs Within Sustainable Horticulture

Beyond their physiological effects, microalgae-derived inputs are increasingly discussed within the framework of sustainable and resource-efficient agriculture. Recent reviews highlight their potential integration into circular bioeconomy models, including nutrient recycling and reduced dependency on synthetic growth regulators [6,11,13]. In greenhouse ornamental systems—where structural stability, uniformity, and resilience are key quality parameters—biologically derived biostimulants may complement conventional fertilization strategies while supporting balanced biomass allocation.

The multi-season consistency observed in the present study reinforces the concept that microalgae-based treatments can function as consistent modulators of structural development under protected cultivation conditions.

### 4.6. Environmental Conditions

Seasonal environmental conditions during the three experimental cycles were characterized using daily meteorological records obtained from the UniSense agrometeorological monitoring system operated by Széchenyi István University (Mosonmagyaróvár, Hungary). UniSense is a university-developed, precision agriculture-oriented IoT platform designed for continuous crop-environment monitoring and decision support. The system integrates multiple atmospheric and radiation sensors and transmits data via NB-IoT communication at short, configurable intervals (typically 10–15 min). For the present study, high-frequency measurements were aggregated into daily values and filtered to the exact 42-day cultivation windows corresponding to each production year.

**Figure 7:**
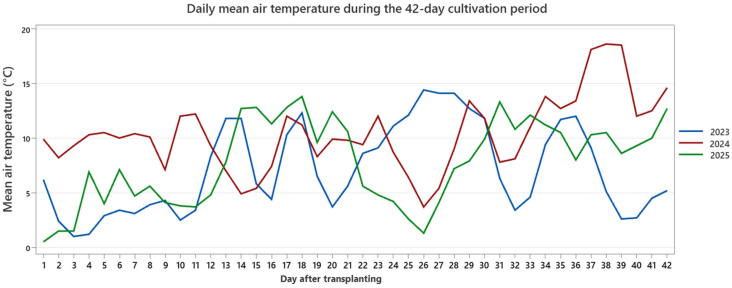
Daily mean air temperature during the 42-day cultivation period (2023–2025). Values represent daily means derived from high-frequency institutional meteorological records and filtered to the defined production windows. Interannual differences illustrate distinct seasonal thermal patterns across the three experimental cycles.

**Figure 8:**
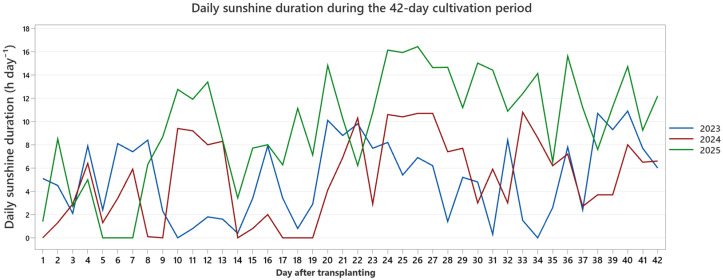
Daily sunshine duration during the 42-day cultivation period (2023–2025). Daily sunshine hours were calculated from institutional meteorological data and restricted to the defined cultivation intervals. Temporal variation reflects differences in light availability among production years.

**Figure 9:**
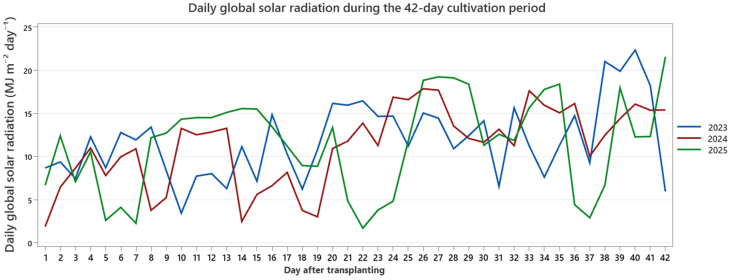
Daily global solar radiation during the 42-day cultivation period (2023–2025). Daily radiation totals (MJ m^−2^ day^−1^) were aggregated from high-resolution meteorological measurements and filtered to the experimental windows. Interannual variability demonstrates differences in radiation intensity during early-season greenhouse production.

The monitored variables included air temperature, sunshine duration, and global solar radiation. Descriptive statistics for each parameter and year are summarized in Table 4, while the temporal dynamics of daily mean air temperature, sunshine duration, and global radiation are presented separately in Figure 7, Figure 8 and Figure 9.

The meteorological station is located ~25 km from the experimental greenhouse within the same regional climatic zone of northwestern Hungary. Although the polytunnel creates a distinct microclimate relative to open-field conditions, the reported variables are intended to describe the macroclimatic thermal and radiation background characterizing regional early-season production. Accordingly, these data are included to contextualize interannual variability between cycles rather than to model greenhouse microclimate directly, as the actual plant environment was governed by greenhouse management. Therefore, these external records are provided for background context only and are not used to mechanistically explain treatment responses.

Across the three years, the environmental profiles were comparable in seasonal timing but differed in magnitude and temporal distribution. During the 2023 cultivation window, mean air temperature was 7.13 °C (range: 1.0–14.4 °C), with moderate fluctuations and cooler early-phase conditions (Figure 7). Daily sunshine duration averaged 5.08 h (0–10.9 h), and mean global solar radiation was 11.86 MJ m^−2^ day^−1^ (3.429–22.317 MJ m^−2^), reflecting variable cloud cover patterns (Figure 7 and Figure 8).

The 2024 production cycle was characterized by generally warmer conditions, with a mean air temperature of 10.38 °C and maximum values reaching 18.6 °C (Figure 7). Sunshine duration remained similar in average magnitude (5.16 h day^−1^), although mid- and late-period peaks were more frequent compared to 2023 (Figure 8). Mean global solar radiation was 11.28 MJ m^−2^ day^−1^, with a narrower overall amplitude than in 2023 (Figure 9).

In contrast, 2025 exhibited a distinct radiation and light profile. While mean air temperature (7.78 °C; range: 0.5–13.8 °C) was comparable to 2023, sunshine duration was markedly higher, averaging 9.73 h day^−1^ and reaching a maximum of 16.44 h (Figure 8). Global solar radiation averaged 11.51 MJ m^−2^ day^−1^, with pronounced peaks during the latter phase of the cultivation window (Figure 9). Across all years, the daily trajectories revealed noticeable short-term fluctuations, including transient cooling periods and episodic radiation peaks, rather than smooth seasonal progression. Collectively, the three production cycles therefore reflect moderately distinct early-season macroclimatic backgrounds based on external station records: a cooler and more variable year (2023), a warmer year with elevated late-period temperatures (2024), and a year characterized by higher light availability and radiation intensity (2025).

Despite these interannual differences in external thermal and radiation patterns, we detected no statistical evidence of year-specific differences in treatment effects. Accordingly, the environmental dataset is provided as background context for the three production cycles and should not be interpreted as a mechanistic explanation of treatment performance, because polytunnel microclimate (notably RH) was not continuously logged. By combining descriptive statistics (Table 4) with daily temporal trajectories (Figure 7, Figure 8 and Figure 9), this environmental characterization improves transparency in reporting background conditions and helps address potential concerns regarding year-specific bias in growth responses. The underlying environmental dataset is provided in Supplementary Material S5.

### 4.7. Destructive Sampling and Growth Measurements

Destructive sampling was conducted once in each growing season, 42 days after transplanting and directly before the commercial sale stage. Within a given season, all plants were harvested on the same day to ensure comparability. Root systems were gently washed to remove adhering substrate particles before further measurements were taken.

The following parameters were recorded:

• Fresh plant mass (g);
• Root mass (g);
• Plant height (cm);
• Root collar diameter (mm).

Dry mass was determined after air-drying plant tissues under ambient laboratory conditions until constant weight. Samples were weighed at 24 h intervals, and drying was considered complete when the change between two consecutive measurements was <0.1% (*w*/*w*). The dry mass value at constant weight was recorded.

Fresh mass was recorded as a handling-related operational measure; however, it was not analyzed further because it closely tracked dry mass and did not change the interpretation of treatment effects. Root length was recorded as an auxiliary measurement, but it was not included among the predefined primary endpoints and therefore was not subjected to GLM-based inference or reported in the Results section.

### 4.8. Statistical Analysis

The dataset was evaluated using a General Linear Model (GLM) framework, with Treatment, Cultivar, and Year included as fixed factors together with their two-way interactions:

Response = Treatment + Cultivar + Year + Treatment × Cultivar + Treatment × Year + Cultivar × Year

Differences were considered statistically significant at *p* < 0.05. Relative percentage changes were calculated from pooled treatment means derived from descriptive statistics across the three experimental seasons (n = 54 per treatment), using the untreated control as the reference value.

Model performance was assessed using the coefficient of determination (R^2^). Data are reported as mean values accompanied by 95% confidence intervals.

Full ANOVA outputs (df, Adj SS, Adj MS, F and *p* for all model terms and interactions) for each response variable are provided in Supplementary Materials S1–S4 (GLM outputs for dry plant mass, root mass, plant height and root collar diameter).

In addition to the primary endpoints reported here (plant height, root collar diameter, dry plant mass, and root mass), fresh plant mass and root length were recorded for operational/monitoring purposes.

These variables were not included in the formal statistical analyses because they were not collected with fully comparable methodology and/or complete coverage across all seasons and showed higher measurement variability. Year and cultivar were treated as fixed factors because the study intentionally compared three specific consecutive production seasons and six commercially relevant cultivars. Inference is therefore focused on repeatability within these defined seasons and genotypes under the tested production system, rather than on generalization to an unbounded population of years or cultivars.

### 4.9. Model Diagnostics and Assumption Checks

To support valid inference from the factorial General Linear Model (GLM), model assumptions were evaluated using standard residual diagnostics, focusing on (i) approximate normality of residuals and (ii) homogeneity of variances across factor levels.

All checks were performed in Minitab^®^ (Version 22.1, 64-bit). Normality testing was conducted using the Ryan–Joiner (RJ) test, which is the default normality test provided in the Minitab “Normality Test” procedure and is widely used as an analogue to Shapiro–Wilk-type normality assessments in applied settings. In addition to formal tests, normal probability plots were inspected because formal normality tests can be overly sensitive in moderately sized datasets, and minor tail deviations may reach statistical significance without materially affecting GLM estimates.

Residual normality was assessed for each response variable using the model residuals (Table 5).

**Table 5:** Normality (Ryan–Joiner) and homogeneity of variance (Levene) tests for GLM residuals.

Variable	RJ Test	Levene	Status
Dry Mass	*p* < 0.01	0.494	Acceptable
Root Mass	0.023	0.344	Acceptable
Plant Height	>0.10	0.031	Mild heteroscedasticity
Root Collar Diameter	>0.10	0.414	Acceptable

For plant height and root collar diameter, RJ-based normality testing did not indicate meaningful departures from normality (*p* > 0.10), and probability plots showed residual points closely following the reference line with only minor deviations at the extremes. Root mass showed a small but detectable deviation from strict normality (*p* = 0.023). Dry mass showed the strongest statistical deviation (*p* < 0.01). Importantly, visual inspection of the corresponding probability plots suggested that these departures were mainly driven by slight tail effects rather than pronounced curvature or systematic structure. Because the experimental design is balanced and fully factorial (Treatment × Cultivar × Year) with equal replication, and because GLM estimates of main effects and interactions are generally robust to modest deviations from normality—particularly when residuals remain approximately symmetric and lack strong outliers—the observed deviations were interpreted as minor and not sufficient to invalidate inference.

Homogeneity of variances was evaluated using Levene’s test across treatment groups for each endpoint. Variance equality was supported for dry mass (*p* = 0.494), root mass (*p* = 0.344), and root collar diameter (*p* = 0.414), indicating no evidence of heteroscedasticity that would bias standard errors for these responses. For plant height, Levene’s test indicated a marginal deviation from strict homoscedasticity (*p* = 0.031). However, the group-level dispersion differences were limited in magnitude and did not suggest extreme variance imbalance. In this context, the height model results were retained, while interpretation emphasized the consistency of direction and the strength of treatment signals across the full dataset rather than reliance on borderline effects. Taken together, the combination of (i) mostly acceptable variance behavior, (ii) only mild residual normality departures, and (iii) a balanced factorial design supports the conclusion that the GLM assumptions were sufficiently met for the purposes of hypothesis testing and effect estimation in this study. Overall, diagnostic evaluation indicates that the factorial GLM provides a reliable statistical basis for assessing treatment, cultivar, and year effects on biomass and structural traits. The assumption checks are therefore reported transparently to document model adequacy and to address potential concerns regarding distributional properties of the endpoints. To further reduce sensitivity to mild departures from normality/variance homogeneity, we prioritize effect-size interpretation (means with 95% CIs) and the consistency of treatment ordering across factors; future work could additionally confirm inference using robust or transformation-based alternatives.

### 4.10. Data Availability and Ethical Considerations

The datasets produced and analyzed during the present study are available from the corresponding author upon reasonable request.

This research did not involve human participants or animal subjects and therefore did not require ethical approval.

Generative AI was used to support English language editing and to improve the structure and readability of the manuscript. The authors reviewed and edited all AI-assisted text and take full responsibility for the content. AI was not used for data generation, statistical analyses, or interpretation.

## 5. Conclusions

This multi-season greenhouse investigation shows that microalgae-based treatments can promote biomass formation and root-linked structural growth in *Pelargonium* cultivated in substrate-based ornamental systems. Across three successive production cycles, we recorded statistically supported increases in dry mass, root mass, and root collar diameter relative to the control, indicating that the responses were not limited to a single season under the tested conditions. In line with aim (i), the greenhouse trials therefore provide evidence for treatment-associated improvements in the measured growth-related traits. With respect to aims (ii)–(iii), responses were directionally consistent across seasons (no statistical evidence of a Treatment × Year interaction under the tested conditions); the largest and most consistent effects were observed in root-linked parameters and their associated shoot outcomes, while response magnitudes varied among cultivars. Importantly, no adverse cultivar-specific responses were observed, suggesting broad compatibility of the tested strains across the evaluated genetic backgrounds. Because the present dataset is morphological and mechanisms were not directly tested, future work should focus on dose–frequency optimization and mechanistic validation under broader commercial scenarios.

### Limitations and Future Work

Future work should (i) quantify flowering traits and market-relevant quality indices alongside vegetative growth, (ii) test performance under reduced fertilization regimes and across additional ornamental species, and (iii) integrate tighter greenhouse microclimate logging (e.g., continuous RH and PAR, and where feasible canopy temperature) to better link environmental dynamics to trait responses.

To connect inputs to outcomes more directly, future trials should also characterize the applied biomass/extract using basic batch-level markers and expand optimization beyond the operationally simple protocol used here (a single application at transplanting and a single dose level) by implementing structured dose–response designs, comparing single versus repeated applications, and evaluating alternative delivery modes (e.g., drench versus fertigation-compatible approaches).

With respect to experimental control under commercial polytunnel conditions, future studies could further strengthen management of edge/gradient effects by applying explicit row/bench blocking and/or planned within-cycle rotation where feasible. In addition, optional quality-control steps such as microbial-load screening (e.g., plating or 16S-based checks) could be incorporated to further support season-to-season comparability and to clarify potential microalgae–bacteria contributions.

Finally, to avoid over-interpreting cross-trait differences, future work should include explicit biomass-allocation analyses (e.g., shoot:root or shoot dry mass:root dry mass ratios) using ratio-based modelling to test whether treatments influence organ partitioning beyond trait-wise effects. Beyond horticultural biostimulant applications, broader circular-economy pathways for microalgae—including model taxa such as *Chlamydomonas reinhardtii* in bioremediation and bioproduct production—also warrant dedicated investigation to connect sustainability routes with greenhouse-relevant plant-performance outcomes.

Taken together, these findings indicate that microalgae applications can improve key structural traits (as measured here) and may contribute to more consistent production outcomes in controlled horticulture. Because mechanical properties and causal mechanisms were not directly measured, interpretation is limited to morphological endpoints; follow-up trials are needed to define the operating window for practical use (e.g., under reduced fertilization, across additional crops, and with commercially compatible application regimes).

## Figures and Tables

**Table 1:** Summary of General Linear Model results for biomass and structural parameters.

Parameter	Treatment *F*	Treatment *p*	Treatment × Year *p*	Treatment × Cultivar *p*	R^2^
Dry mass	28.31	<0.001	0.915	<0.001	0.6278
Root mass	14.15	<0.001	0.197	<0.001	0.7834
Root collar diameter	19.00	<0.001	0.568	0.006	0.8925
Plant height	8.90	<0.001	1.000	<0.001	0.7481

*F*-values correspond to treatment main effects.

**Table 2:** Microalgae strains selected from the Mosonmagyaróvár Algal Culture Collection (MACC).

ID	MACC Code	Algae Name	Algae Type
T1	612	*Nostoc piscinale*	Cyanobacterium (blue-green algae)
T2	922	*Chlorella vulgaris*	Green microalgae
K	Control	N/A	Untreated control

**Table 3:** *Pelargonium* cultivars evaluated in the greenhouse experiment.

ID	Botanical Type	Commercial Name
A	*Zonale*	*Andria™*
B	*Zonale*	*Savanna Really Red™*
C	*Peltatum*	*Great Balls of Fire™*
D	*Peltatum*	*Atlantic Dark Red™*
E	*Lateripes*	*Classic Single Villa Paris Lilac™*
F	*Lateripes*	*Classic Single Villa Dresden™*

**Table 4:** Descriptive Statistics of environmental parameters.

Variable	Year	N	Mean	SE Mean	StDev	Min	Max
T [°C] Average	2023	42	7.12857	0.629421	4.07911	1	14.4
	2024	42	10.3833	0.521745	3.38129	3.7	18.6
	2025	42	7.78333	0.594847	3.85505	0.5	13.8
Daily sunshine duration (h)	2023	42	5.07857	0.532169	3.44885	0	10.9
	2024	42	5.15714	0.566825	3.67345	0	10.8
	2025	42	9.73855	0.728675	4.72235	0	16.44
Daily global solar radiation (MJ/M^2^)	2023	42	11.856	0.66732	4.32473	3.429	22.317
	2024	42	11.2768	0.694955	4.50382	1.876	17.837
	2025	42	11.5118	0.840321	5.4459	1.688	21.534

## Data Availability

The original contributions presented in this study are included in the article/Supplementary Material. Further inquiries can be directed to the corresponding authors.

## Supplementary Materials

The following supporting information can be downloaded at: https://www.mdpi.com/article/10.3390/plants15050803/s1. Supplementary Materials S1: Dry Plant Mass(g)—GLM + Probability Plot + Test for Equal Variances.pdf; Supplementary Materials S2: Root Mass(g)—GLM + Probability Plot + Test for Equal Variances.pdf; Supplementary Materials S3: Plant Height (cm)—GLM + Probability Plot + Test for Equal Variances.pdf; Supplementary Materials S4: Root Collar Diameter (mm)—GLM + Probability Plot + Test for Equal Variances.pdf; Supplementary Materials S5: Environmental data.xlsx; Supplementary Materials S6: Descriptive Statistics collection.pdf; Supplementary Materials S7: Base data for End-Point Measurements.xlsx; Supplementary Materials S8: Cultivar-level effect size.xlsx.

## Abbreviations

The following abbreviations are used in this manuscript:

GLM	General Linear Model
MACC	Mosonmagyaróvár Algal Culture Collection
CI	Confidence Interval
CV	Coefficient of Variation
MSE	Mean Square Error
MS	Mean Square
Adj MS	Adj MS—adjusted mean square
SS	Sum of squares
Adj SS	Adj SS—adjusted sum of squares
RJ	Ryan–Joiner (normality) test
EC	Electrical conductivity
IoT	Internet of Things
NB-IoT	Narrowband Internet of Things
df	Degrees of freedom
