# Supplementary material for: Microalgae-Based Biostimulants Improve Biomass Production and Root-Linked Performance Stability in Pelargonium: A Three-Year Greenhouse Study
Source: Plants (Basel). 2026 Mar 5;15(5):803. doi: 10.3390/plants15050803 (PMC12986712; doi:10.3390/plants15050803)
Supplement: Supplementary file 1 [file plants-15-00803-s001.zip › S1 - Dry Plant Mass(g) - GLM+Probability Plot+Test for Equal Variances.pdf]

Dry Plant Mass(g) - GLM+Probability Plot+Test for Equal Variances

Method

Factor coding	(-1; 0; +1)

Factor Information

Factor	Type	Levels	Values
Treatment	Fixed	3	K; T1; T2
CultivarCode	Fixed	6	A; B; C; D; E; F
Year	Fixed	3	2023; 2024; 2025

Analysis of Variance

Source	DF	Adj SS	Adj MS	F-Value	P-Value
Treatment	2	37,131	18,5657	28,31	0,000
CultivarCode	5	39,991	7,9981	12,20	0,000
Year	2	29,625	14,8127	22,59	0,000
Treatment*CultivarCode	10	28,043	2,8043	4,28	0,000
Treatment*Year	4	0,630	0,1576	0,24	0,915
CultivarCode*Year	10	6,125	0,6125	0,93	0,504
Error	128	83,928	0,6557		
Lack-of-Fit	20	8,510	0,4255	0,61	0,898
Pure Error	108	75,419	0,6983		
Total	161	225,474			

Model Summary

S	R-sq	R-sq(adj)	R-sq(pred)
0,809747	62,78%	53,18%	40,38%

Coefficients

Term	Coef	SE Coef	T-Value	P-Value	VIF
Constant	4,1431	0,0636	65,12	0,000	
Treatment					
K	-0,6154	0,0900	-6,84	0,000	1,33
T1	0,0631	0,0900	0,70	0,484	1,33
CultivarCode					
A	-0,669	0,142	-4,70	0,000	1,67
B	-0,128	0,142	-0,90	0,369	1,67
C	0,640	0,142	4,50	0,000	1,67
D	0,198	0,142	1,39	0,167	1,67
E	0,512	0,142	3,60	0,000	1,67
Year					
2023	0,0289	0,0900	0,32	0,749	1,33
2024	0,5087	0,0900	5,65	0,000	1,33
Treatment*CultivarCode					
K A	-0,014	0,201	-0,07	0,944	2,22
K B	-0,477	0,201	-2,37	0,019	2,22
K C	-0,479	0,201	-2,38	0,019	2,22
K D	0,408	0,201	2,03	0,045	2,22
K E	0,160	0,201	0,79	0,428	2,22

T1 A	0,329	0,201	1,64	0,104	2,22
T1 B	0,289	0,201	1,44	0,154	2,22
T1 C	0,820	0,201	4,08	0,000	2,22
T1 D	-0,259	0,201	-1,29	0,200	2,22
T1 E	-0,752	0,201	-3,74	0,000	2,22
Treatment*Year					
K 2023	-0,057	0,127	-0,45	0,657	1,78
K 2024	-0,042	0,127	-0,33	0,742	1,78
T1 2023	0,084	0,127	0,66	0,512	1,78
T1 2024	-0,037	0,127	-0,29	0,770	1,78
CultivarCode*Year					
A 2023	-0,159	0,201	-0,79	0,432	2,22
A 2024	-0,005	0,201	-0,02	0,980	2,22
B 2023	0,012	0,201	0,06	0,953	2,22
B 2024	-0,068	0,201	-0,34	0,736	2,22
C 2023	0,071	0,201	0,35	0,724	2,22
C 2024	0,097	0,201	0,48	0,631	2,22
D 2023	-0,203	0,201	-1,01	0,315	2,22
D 2024	0,139	0,201	0,69	0,489	2,22
E 2023	0,527	0,201	2,62	0,010	2,22
E 2024	-0,242	0,201	-1,20	0,231	2,22

Regression Equation

Dry Plant Mass (g) = 4,1431 - 0,6154 Treatment_K + 0,0631 Treatment_T1 + 0,5522 Treatment_T2
- 0,669 CultivarCode_A - 0,128 CultivarCode_B + 0,640 CultivarCode_C
+ 0,198 CultivarCode_D + 0,512 CultivarCode_E - 0,553 CultivarCode_F
+ 0,0289 Year_2023 + 0,5087 Year_2024 - 0,5376 Year_2025
- 0,014 Treatment*CultivarCode_K A - 0,477 Treatment*CultivarCode_K B
- 0,479 Treatment*CultivarCode_K C + 0,408 Treatment*CultivarCode_K D
+ 0,160 Treatment*CultivarCode_K E + 0,403 Treatment*CultivarCode_K F
+ 0,329 Treatment*CultivarCode_T1 A + 0,289 Treatment*CultivarCode_T1 B
+ 0,820 Treatment*CultivarCode_T1 C - 0,259 Treatment*CultivarCode_T1 D
- 0,752 Treatment*CultivarCode_T1 E - 0,427 Treatment*CultivarCode_T1 F
- 0,315 Treatment*CultivarCode_T2 A + 0,189 Treatment*CultivarCode_T2 B
- 0,341 Treatment*CultivarCode_T2 C - 0,149 Treatment*CultivarCode_T2 D
+ 0,592 Treatment*CultivarCode_T2 E + 0,024 Treatment*CultivarCode_T2 F
- 0,057 Treatment*Year_K 2023 - 0,042 Treatment*Year_K 2024
+ 0,099 Treatment*Year_K 2025 + 0,084 Treatment*Year_T1 2023
- 0,037 Treatment*Year_T1 2024 - 0,046 Treatment*Year_T1 2025
- 0,027 Treatment*Year_T2 2023 + 0,079 Treatment*Year_T2 2024
- 0,052 Treatment*Year_T2 2025 - 0,159 CultivarCode*Year_A 2023
- 0,005 CultivarCode*Year_A 2024 + 0,164 CultivarCode*Year_A 2025
+ 0,012 CultivarCode*Year_B 2023 - 0,068 CultivarCode*Year_B 2024
+ 0,056 CultivarCode*Year_B 2025 + 0,071 CultivarCode*Year_C 2023
+ 0,097 CultivarCode*Year_C 2024 - 0,168 CultivarCode*Year_C 2025
- 0,203 CultivarCode*Year_D 2023 + 0,139 CultivarCode*Year_D 2024
+ 0,064 CultivarCode*Year_D 2025 + 0,527 CultivarCode*Year_E 2023
- 0,242 CultivarCode*Year_E 2024 - 0,285 CultivarCode*Year_E 2025
- 0,248 CultivarCode*Year_F 2023 + 0,079 CultivarCode*Year_F 2024
+ 0,169 CultivarCode*Year_F 2025

Fits and Diagnostics for Unusual Observations

Obs	Dry Plant Mass (g)	Fit	Resid	Std Resid	
12	2,200	3,920	-1,720	-2,39	R
17	5,300	3,091	2,209	3,07	R
19	1,800	3,732	-1,932	-2,68	R

22	6,500	3,732	2,768	3,85	R
59	5,900	4,196	1,704	2,37	R
73	2,600	4,252	-1,652	-2,30	R
76	5,900	4,252	1,648	2,29	R
113	5,000	3,098	1,902	2,64	R

R Large residual

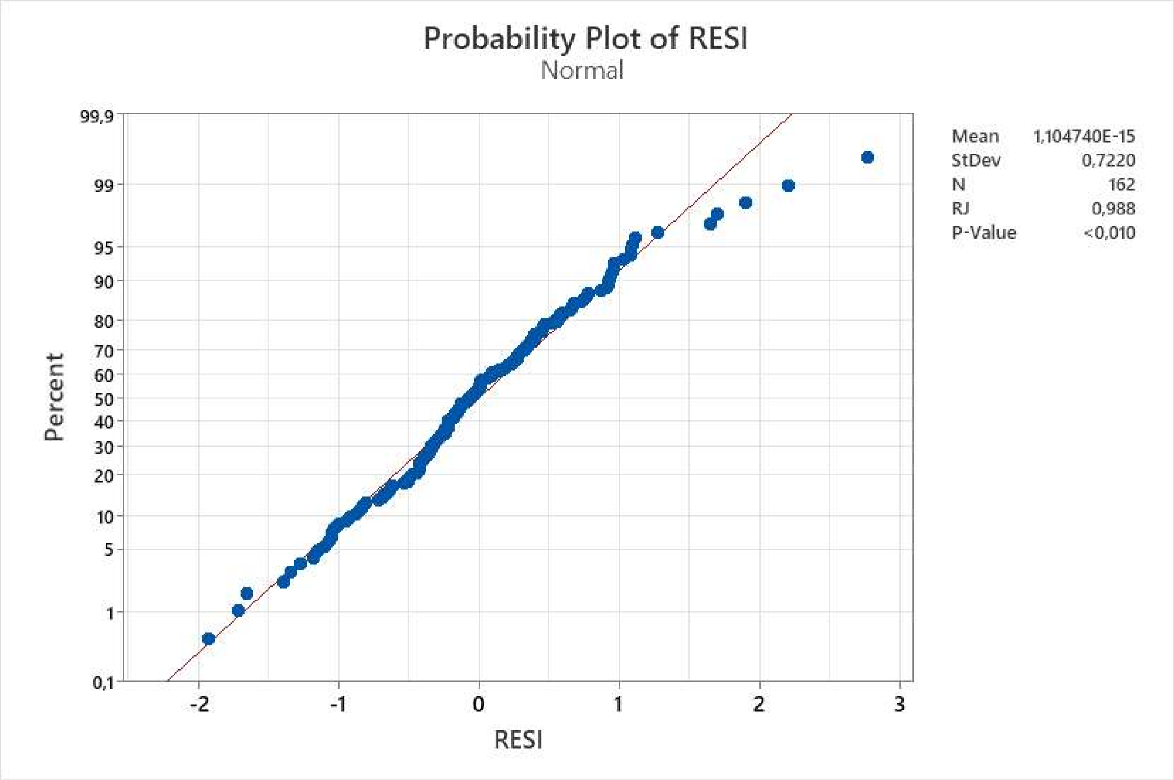

Method

Null hypothesis	All variances are equal
Alternative hypothesis	At least one variance is different
Significance level	α = 0,05

95% Bonferroni Confidence Intervals for Standard Deviations

Treatment	N	StDev	CI
K	54	0,99932	(0,770075; 1,35698)
T1	54	1,14078	(0,929600; 1,46488)
T2	54	1,11965	(0,907332; 1,44574)

Individual confidence level = 98,3333%

Tests

Method	Test Statistic	P-Value
Multiple comparisons	—	0,622
Levene	0,71	0,494

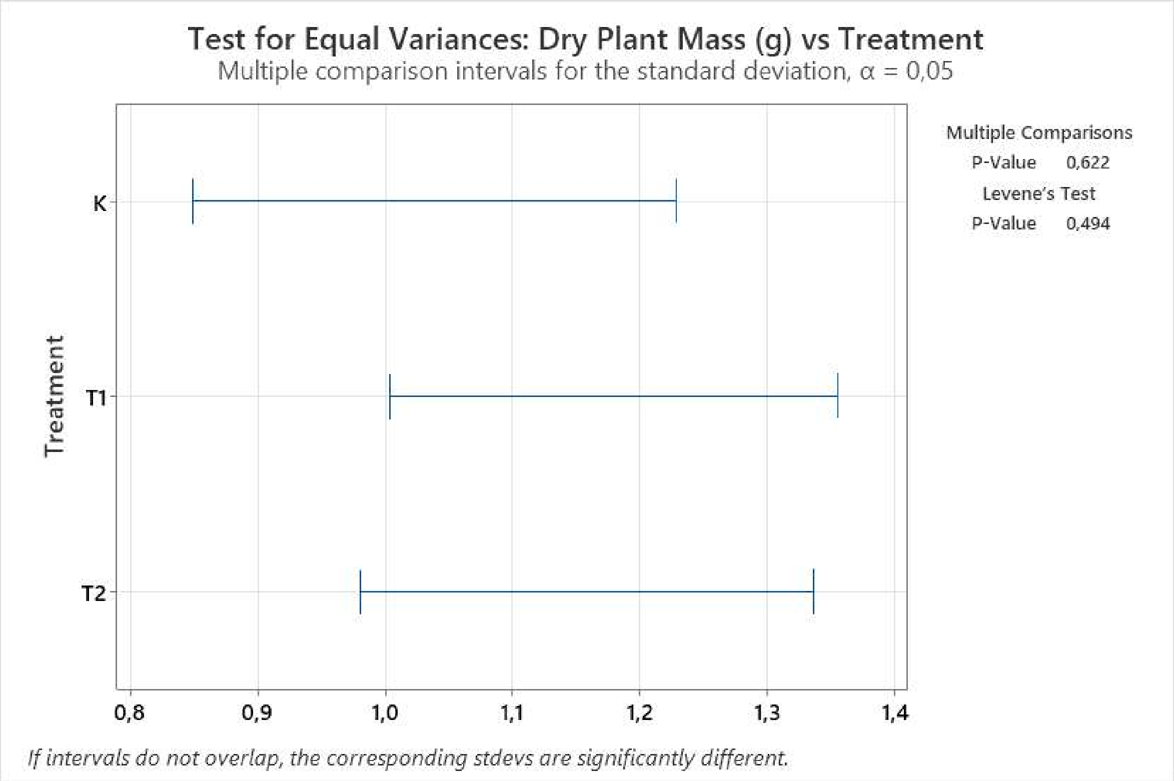
Test for Equal Variances: Dry Plant Mass (g) vs Treatment
Multiple comparison intervals for the standard deviation, α = 0,05
Multiple Comparisons
P-Value 0,622
Levene's Test
P-Value 0,494
Treatment
K
T1
T2
0,8
0,9
1,0
1,1
1,2
1,3
1,4
If intervals do not overlap, the corresponding stdevs are significantly different.
