# Supplementary material for: Microalgae-Based Biostimulants Improve Biomass Production and Root-Linked Performance Stability in Pelargonium: A Three-Year Greenhouse Study
Source: Plants (Basel). 2026 Mar 5;15(5):803. doi: 10.3390/plants15050803 (PMC12986712; doi:10.3390/plants15050803)
Supplement: Supplementary file 1 [file plants-15-00803-s001.zip › S2 - Root Mass(g) - GLM+Probability Plot+Test for Equal Variances.pdf]

Root Mass (g) - GLM+Probability Plot+Test for Equal Variances

Method

Factor coding	(-1; 0; +1)

Factor Information

Factor	Type	Levels	Values
Treatment	Fixed	3	K; T1; T2
CultivarCode	Fixed	6	A; B; C; D; E; F
Year	Fixed	3	2023; 2024; 2025

Analysis of Variance

Source	DF	Adj SS	Adj MS	F-Value	P-Value
Treatment	2	4,4245	2,2123	14,15	0,000
CultivarCode	5	52,8400	10,5680	67,57	0,000
Year	2	6,0135	3,0067	19,22	0,000
Treatment*CultivarCode	10	5,9603	0,5960	3,81	0,000
Treatment*Year	4	0,9570	0,2393	1,53	0,197
CultivarCode*Year	10	2,1900	0,2190	1,40	0,187
Error	128	20,0188	0,1564		
Lack-of-Fit	20	1,1936	0,0597	0,34	0,996
Pure Error	108	18,8253	0,1743		
Total	161	92,4041			

Model Summary

S	R-sq	R-sq(adj)	R-sq(pred)
0,395471	78,34%	72,75%	65,30%

Coefficients

Term	Coef	SE Coef	T-Value	P-Value	VIF
Constant	1,7414	0,0311	56,04	0,000	
Treatment					
K	-0,2332	0,0439	-5,31	0,000	1,33
T1	0,1033	0,0439	2,35	0,020	1,33
CultivarCode					
A	0,6490	0,0695	9,34	0,000	1,67
B	0,5898	0,0695	8,49	0,000	1,67
C	0,2283	0,0695	3,29	0,001	1,67
D	0,0172	0,0695	0,25	0,805	1,67
E	-0,6114	0,0695	-8,80	0,000	1,67
Year					
2023	-0,0821	0,0439	-1,87	0,064	1,33
2024	0,2660	0,0439	6,05	0,000	1,33
Treatment*CultivarCode					
K A	-0,3638	0,0983	-3,70	0,000	2,22
K B	-0,0468	0,0983	-0,48	0,635	2,22
K C	0,1336	0,0983	1,36	0,176	2,22
K D	0,1258	0,0983	1,28	0,203	2,22
K E	0,1188	0,0983	1,21	0,229	2,22

T1 A	0,4375	0,0983	4,45	0,000	2,22
T1 B	-0,2966	0,0983	-3,02	0,003	2,22
T1 C	-0,1273	0,0983	-1,30	0,197	2,22
T1 D	0,0582	0,0983	0,59	0,555	2,22
T1 E	-0,1188	0,0983	-1,21	0,229	2,22
Treatment*Year					
K 2023	0,0428	0,0621	0,69	0,492	1,78
K 2024	-0,0520	0,0621	-0,84	0,404	1,78
T1 2023	-0,1453	0,0621	-2,34	0,021	1,78
T1 2024	0,0949	0,0621	1,53	0,129	1,78
CultivarCode*Year					
A 2023	-0,0005	0,0983	-0,01	0,996	2,22
A 2024	0,0847	0,0983	0,86	0,390	2,22
B 2023	0,1410	0,0983	1,43	0,154	2,22
B 2024	0,0284	0,0983	0,29	0,773	2,22
C 2023	-0,0453	0,0983	-0,46	0,645	2,22
C 2024	0,0421	0,0983	0,43	0,669	2,22
D 2023	-0,2675	0,0983	-2,72	0,007	2,22
D 2024	0,1199	0,0983	1,22	0,225	2,22
E 2023	0,1421	0,0983	1,45	0,151	2,22
E 2024	-0,1516	0,0983	-1,54	0,125	2,22

Regression Equation

Root Mass (g) = 1,7414 - 0,2332 Treatment_K + 0,1033 Treatment_T1 + 0,1299 Treatment_T2
+ 0,6490 CultivarCode_A + 0,5898 CultivarCode_B + 0,2283 CultivarCode_C
+ 0,0172 CultivarCode_D - 0,6114 CultivarCode_E - 0,8728 CultivarCode_F
- 0,0821 Year_2023 + 0,2660 Year_2024 - 0,1840 Year_2025
- 0,3638 Treatment*CultivarCode_K A - 0,0468 Treatment*CultivarCode_K B
+ 0,1336 Treatment*CultivarCode_K C + 0,1258 Treatment*CultivarCode_K D
+ 0,1188 Treatment*CultivarCode_K E + 0,0325 Treatment*CultivarCode_K F
+ 0,4375 Treatment*CultivarCode_T1 A - 0,2966 Treatment*CultivarCode_T1 B
- 0,1273 Treatment*CultivarCode_T1 C + 0,0582 Treatment*CultivarCode_T1 D
- 0,1188 Treatment*CultivarCode_T1 E + 0,0471 Treatment*CultivarCode_T1 F
- 0,0736 Treatment*CultivarCode_T2 A + 0,3434 Treatment*CultivarCode_T2 B
- 0,0062 Treatment*CultivarCode_T2 C - 0,1840 Treatment*CultivarCode_T2 D
+ 0,0001 Treatment*CultivarCode_T2 E - 0,0796 Treatment*CultivarCode_T2 F
+ 0,0428 Treatment*Year_K 2023 - 0,0520 Treatment*Year_K 2024
+ 0,0091 Treatment*Year_K 2025 - 0,1453 Treatment*Year_T1 2023
+ 0,0949 Treatment*Year_T1 2024 + 0,0504 Treatment*Year_T1 2025
+ 0,1025 Treatment*Year_T2 2023 - 0,0429 Treatment*Year_T2 2024
- 0,0596 Treatment*Year_T2 2025 - 0,0005 CultivarCode*Year_A 2023
+ 0,0847 CultivarCode*Year_A 2024 - 0,0842 CultivarCode*Year_A 2025
+ 0,1410 CultivarCode*Year_B 2023 + 0,0284 CultivarCode*Year_B 2024
- 0,1694 CultivarCode*Year_B 2025 - 0,0453 CultivarCode*Year_C 2023
+ 0,0421 CultivarCode*Year_C 2024 + 0,0032 CultivarCode*Year_C 2025
- 0,2675 CultivarCode*Year_D 2023 + 0,1199 CultivarCode*Year_D 2024
+ 0,1477 CultivarCode*Year_D 2025 + 0,1421 CultivarCode*Year_E 2023
- 0,1516 CultivarCode*Year_E 2024 + 0,0095 CultivarCode*Year_E 2025
+ 0,0302 CultivarCode*Year_F 2023 - 0,1235 CultivarCode*Year_F 2024
+ 0,0932 CultivarCode*Year_F 2025

Fits and Diagnostics for Unusual Observations

Obs	Root Mass (g)	Fit	Resid	Std Resid	
17	1,900	0,822	1,078	3,07	R
46	3,310	2,153	1,157	3,29	R
48	3,910	2,966	0,944	2,69	R

50	1,230	2,051	-0,821	-2,34	R
51	2,170	2,966	-0,796	-2,26	R
100	3,310	2,294	1,016	2,89	R

R Large residual

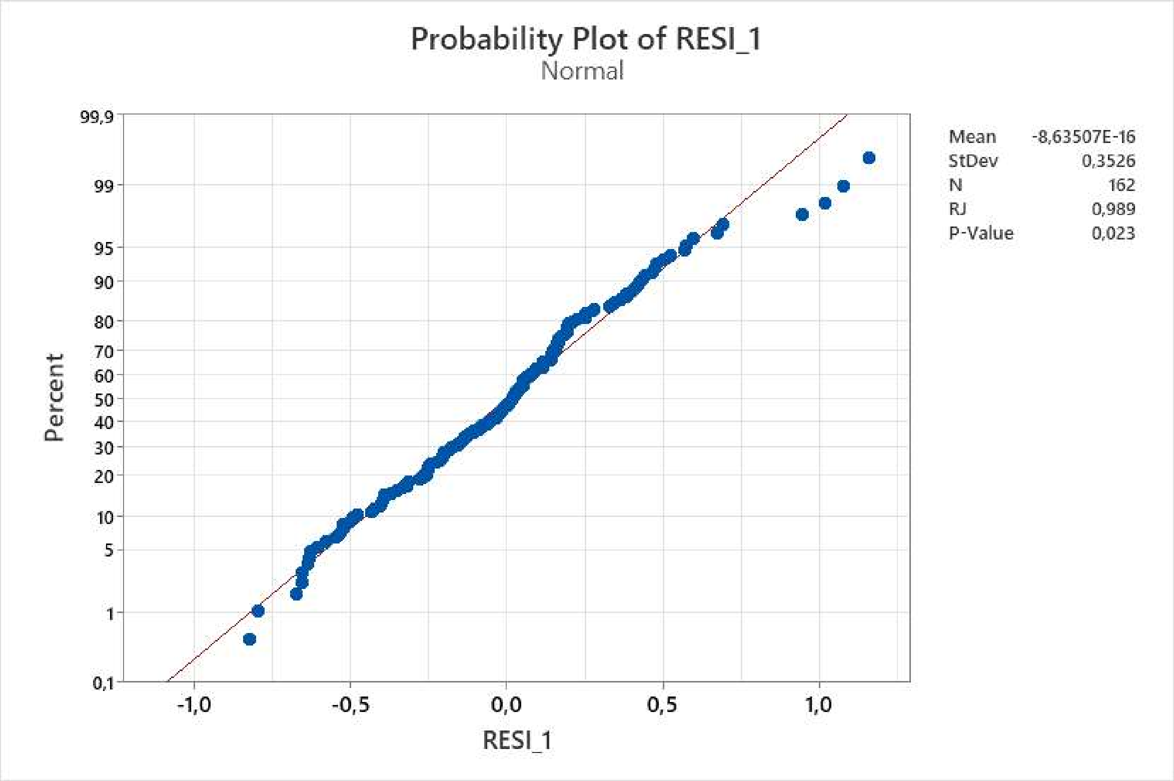

Method

Null hypothesis	All variances are equal
Alternative hypothesis	At least one variance is different
Significance level	α = 0,05

95% Bonferroni Confidence Intervals for Standard Deviations

Treatment	N	StDev	CI
K	54	0,648645	(0,500234; 0,88010)
T1	54	0,798951	(0,652850; 1,02311)
T2	54	0,775196	(0,630455; 0,99738)

Individual confidence level = 98,3333%

Tests

Method	Test Statistic	P-Value
Multiple comparisons	—	0,305
Levene	1,07	0,344

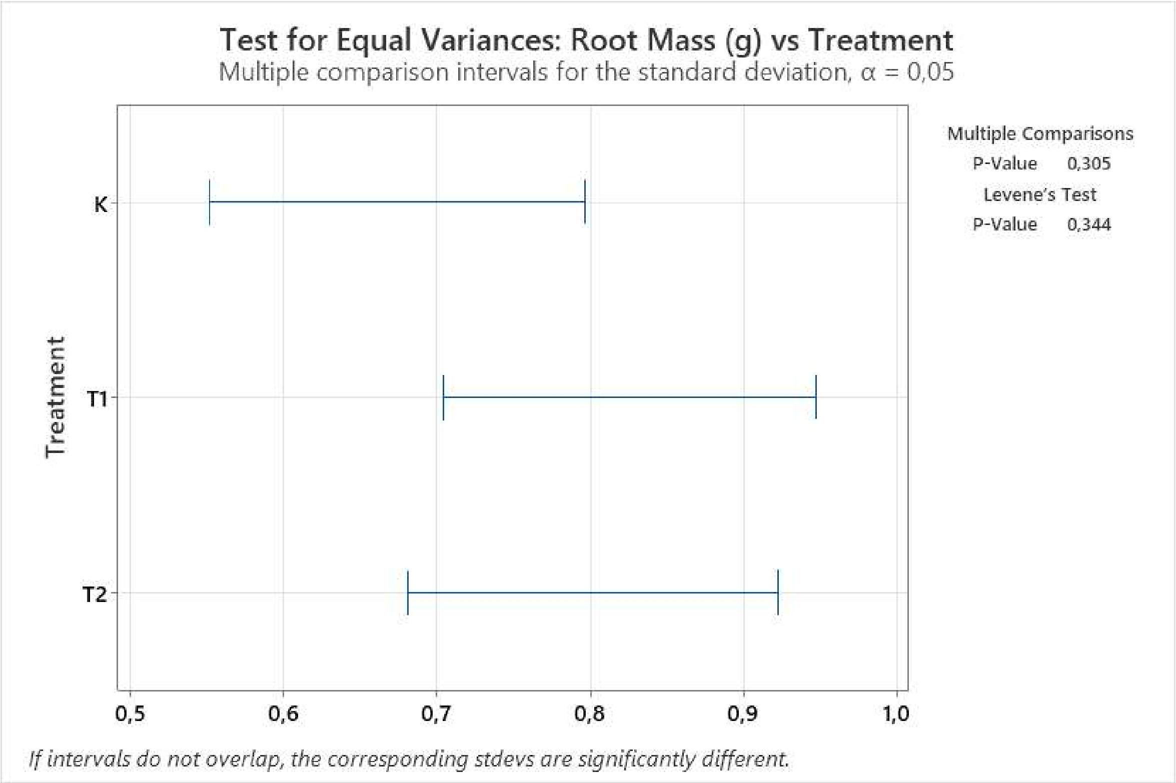
Test for Equal Variances: Root Mass (g) vs Treatment
Multiple comparison intervals for the standard deviation, α = 0,05
Multiple Comparisons
P-Value 0,305
Levene's Test
P-Value 0,344
K
T1
T2
Treatment
0,5
0,6
0,7
0,8
0,9
1,0
If intervals do not overlap, the corresponding stdevs are significantly different.
