# Supplementary material for: Microalgae-Based Biostimulants Improve Biomass Production and Root-Linked Performance Stability in Pelargonium: A Three-Year Greenhouse Study
Source: Plants (Basel). 2026 Mar 5;15(5):803. doi: 10.3390/plants15050803 (PMC12986712; doi:10.3390/plants15050803)
Supplement: Supplementary file 1 [file plants-15-00803-s001.zip › S3 - Plant Height (cm) - GLM+Probability Plot+Test for Equal Variances.pdf]

Plant Height (cm) - GLM+Probability Plot+Test for Equal Variances - Corr.

Method

Factor coding (-1; 0; +1)

Factor Information

Factor	Type	Levels	Values
Treatment	Fixed	3	K; T1; T2
CultivarCode	Fixed	6	A; B; C; D; E; F
Year	Fixed	3	2023; 2024; 2025

Analysis of Variance

Source	DF	Adj SS	Adj MS	F-Value	P-Value
Treatment	2	141,74	70,872	8,90	0,000
CultivarCode	5	2521,26	504,252	63,32	0,000
Year	2	24,01	12,005	1,51	0,225
Treatment*CultivarCode	10	305,48	30,548	3,84	0,000
Treatment*Year	4	0,35	0,087	0,01	1,000
CultivarCode*Year	10	33,67	3,367	0,42	0,933
Error	128	1019,29	7,963		
Lack-of-Fit	20	89,21	4,460	0,52	0,954
Pure Error	108	930,09	8,612		
Total	161	4045,81			

Model Summary

S	R-sq	R-sq(adj)	R-sq(pred)
2,82192	74,81%	68,31%	59,64%

Coefficients

Term	Coef	SE Coef	T-Value	P-Value	VIF
Constant	24,226	0,222	109,27	0,000	
Treatment					
K	-1,313	0,314	-4,19	0,000	1,33
T1	0,796	0,314	2,54	0,012	1,33
CultivarCode					
A	-3,919	0,496	-7,90	0,000	1,67
B	-4,107	0,496	-8,29	0,000	1,67
C	-1,111	0,496	-2,24	0,027	1,67
D	-1,522	0,496	-3,07	0,003	1,67
E	5,959	0,496	12,02	0,000	1,67
Year					
2023	-0,544	0,314	-1,74	0,085	1,33
2024	0,272	0,314	0,87	0,387	1,33
Treatment*CultivarCode					
K A	0,317	0,701	0,45	0,652	2,22
K B	0,639	0,701	0,91	0,364	2,22
K C	-1,913	0,701	-2,73	0,007	2,22
K D	-0,557	0,701	-0,80	0,428	2,22
K E	-0,761	0,701	-1,09	0,280	2,22

T1 A	0,341	0,701	0,49	0,628	2,22
T1 B	0,552	0,701	0,79	0,433	2,22
T1 C	1,311	0,701	1,87	0,064	2,22
T1 D	0,611	0,701	0,87	0,385	2,22
T1 E	-2,370	0,701	-3,38	0,001	2,22
Treatment*Year					
K 2023	-0,074	0,443	-0,17	0,868	1,78
K 2024	0,037	0,443	0,08	0,934	1,78
T1 2023	-0,011	0,443	-0,03	0,980	1,78
T1 2024	0,006	0,443	0,01	0,990	1,78
CultivarCode*Year					
A 2023	-0,285	0,701	-0,41	0,685	2,22
A 2024	0,143	0,701	0,20	0,839	2,22
B 2023	-1,085	0,701	-1,55	0,124	2,22
B 2024	0,543	0,701	0,77	0,440	2,22
C 2023	-0,115	0,701	-0,16	0,870	2,22
C 2024	0,057	0,701	0,08	0,935	2,22
D 2023	0,174	0,701	0,25	0,804	2,22
D 2024	-0,087	0,701	-0,12	0,901	2,22
E 2023	0,248	0,701	0,35	0,724	2,22
E 2024	-0,124	0,701	-0,18	0,860	2,22

Regression Equation

Plant Height (cm) = 24,226 - 1,313 Treatment_K + 0,796 Treatment_T1 + 0,517 Treatment_T2
- 3,919 CultivarCode_A - 4,107 CultivarCode_B - 1,111 CultivarCode_C
- 1,522 CultivarCode_D + 5,959 CultivarCode_E + 4,700 CultivarCode_F
- 0,544 Year_2023 + 0,272 Year_2024 + 0,272 Year_2025
+ 0,317 Treatment*CultivarCode_K A + 0,639 Treatment*CultivarCode_K B
- 1,913 Treatment*CultivarCode_K C - 0,557 Treatment*CultivarCode_K D
- 0,761 Treatment*CultivarCode_K E + 2,276 Treatment*CultivarCode_K F
+ 0,341 Treatment*CultivarCode_T1 A + 0,552 Treatment*CultivarCode_T1 B
+ 1,311 Treatment*CultivarCode_T1 C + 0,611 Treatment*CultivarCode_T1 D
- 2,370 Treatment*CultivarCode_T1 E - 0,444 Treatment*CultivarCode_T1 F
- 0,657 Treatment*CultivarCode_T2 A - 1,191 Treatment*CultivarCode_T2 B
+ 0,602 Treatment*CultivarCode_T2 C - 0,054 Treatment*CultivarCode_T2 D
+ 3,131 Treatment*CultivarCode_T2 E - 1,831 Treatment*CultivarCode_T2 F
- 0,074 Treatment*Year_K 2023 + 0,037 Treatment*Year_K 2024
+ 0,037 Treatment*Year_K 2025 - 0,011 Treatment*Year_T1 2023
+ 0,006 Treatment*Year_T1 2024 + 0,006 Treatment*Year_T1 2025
+ 0,085 Treatment*Year_T2 2023 - 0,043 Treatment*Year_T2 2024
- 0,043 Treatment*Year_T2 2025 - 0,285 CultivarCode*Year_A 2023
+ 0,143 CultivarCode*Year_A 2024 + 0,143 CultivarCode*Year_A 2025
- 1,085 CultivarCode*Year_B 2023 + 0,543 CultivarCode*Year_B 2024
+ 0,543 CultivarCode*Year_B 2025 - 0,115 CultivarCode*Year_C 2023
+ 0,057 CultivarCode*Year_C 2024 + 0,057 CultivarCode*Year_C 2025
+ 0,174 CultivarCode*Year_D 2023 - 0,087 CultivarCode*Year_D 2024
- 0,087 CultivarCode*Year_D 2025 + 0,248 CultivarCode*Year_E 2023
- 0,124 CultivarCode*Year_E 2024 - 0,124 CultivarCode*Year_E 2025
+ 1,063 CultivarCode*Year_F 2023 - 0,531 CultivarCode*Year_F 2024
- 0,531 CultivarCode*Year_F 2025

Fits and Diagnostics for Unusual Observations

Obs	Plant Height (cm)	Fit	Resid	Std Resid	
3	28,50	33,62	-5,12	-2,04	R
22	24,50	19,16	5,34	2,13	R

51	12,50	17,90	-5,40	-2,15	R
52	10,50	17,74	-7,24	-2,89	R
54	26,00	17,90	8,10	3,23	R
105	15,00	20,22	-5,22	-2,08	R
159	15,00	20,22	-5,22	-2,08	R

R Large residual

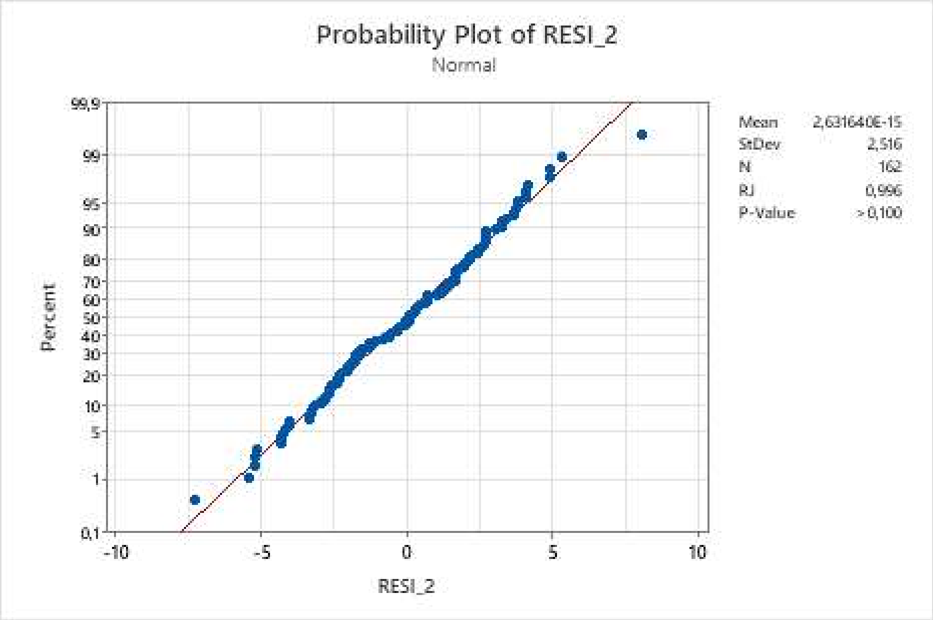

Method

Null hypothesis	All variances are equal
Alternative hypothesis	At least one variance is different
Significance level	α = 0,05

95% Bonferroni Confidence Intervals for Standard Deviations

Treatment	N	StDev	CI
K	54	5,30251	(4,44860; 6,61353)
T1	54	3,60229	(2,98592; 4,54749)
T2	54	5,70688	(4,52884; 7,52497)

Individual confidence level = 98,3333%

Tests

Method	Test Statistic	P-Value
Multiple comparisons	—	0,006
Levene	3,57	0,031

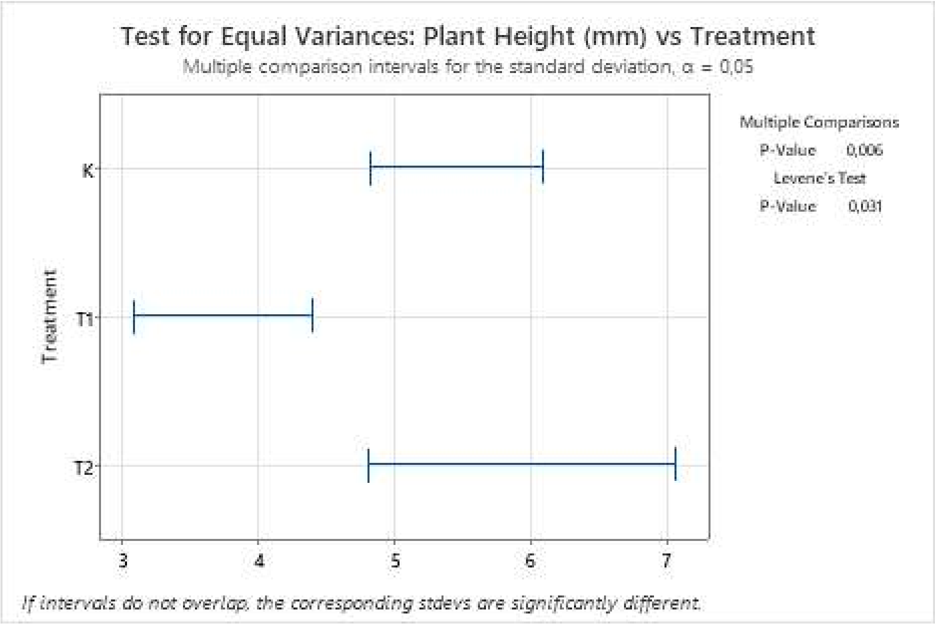
Test for Equal Variances: Plant Height (mm) vs Treatment
Multiple comparison intervals for the standard deviation, α = 0,05
Multiple Comparisons
P-Value 0,006
Levene's Test
P-Value 0,031
Treatment
K
T1
T2
3
4
5
6
7
If intervals do not overlap, the corresponding stdevs are significantly different.
