# Supplementary material for: Microalgae-Based Biostimulants Improve Biomass Production and Root-Linked Performance Stability in Pelargonium: A Three-Year Greenhouse Study
Source: Plants (Basel). 2026 Mar 5;15(5):803. doi: 10.3390/plants15050803 (PMC12986712; doi:10.3390/plants15050803)

Root Collar Diameter (mm) - GLM+Probability Plot+Test for Equal Varianc...

Method

Factor coding	(-1; 0; +1)

Factor Information

Factor	Type	Levels	Values
Treatment	Fixed	3	K; T1; T2
CultivarCode	Fixed	6	A; B; C; D; E; F
Year	Fixed	3	2023; 2024; 2025

Analysis of Variance

Source	DF	Adj SS	Adj MS	F-Value	P-Value
Treatment	2	33,81	16,906	19,00	0,000
CultivarCode	5	684,34	136,869	153,80	0,000
Year	2	128,60	64,298	72,25	0,000
Treatment*CultivarCode	10	23,51	2,351	2,64	0,006
Treatment*Year	4	2,63	0,657	0,74	0,568
CultivarCode*Year	10	72,93	7,293	8,20	0,000
Error	128	113,91	0,890		
Lack-of-Fit	20	4,04	0,202	0,20	1,000
Pure Error	108	109,87	1,017		
Total	161	1059,72			

Model Summary

S	R-sq	R-sq(adj)	R-sq(pred)
0,943344	89,25%	86,48%	82,78%

Coefficients

Term	Coef	SE Coef	T-Value	P-Value	VIF
Constant	7,1356	0,0741	96,28	0,000	
Treatment					
K	-0,575	0,105	-5,49	0,000	1,33
T1	0,033	0,105	0,31	0,754	1,33
CultivarCode					
A	2,181	0,166	13,16	0,000	1,67
B	2,992	0,166	18,05	0,000	1,67
C	0,063	0,166	0,38	0,707	1,67
D	-0,486	0,166	-2,93	0,004	1,67
E	-2,128	0,166	-12,84	0,000	1,67
Year					
2023	-0,036	0,105	-0,35	0,731	1,33
2024	1,109	0,105	10,58	0,000	1,33
Treatment*CultivarCode					
K A	-0,197	0,234	-0,84	0,403	2,22
K B	-0,491	0,234	-2,10	0,038	2,22
K C	-0,025	0,234	-0,11	0,915	2,22
K D	0,293	0,234	1,25	0,213	2,22
K E	-0,067	0,234	-0,29	0,776	2,22

T1 A	0,516	0,234	2,20	0,029	2,22
T1 B	-0,454	0,234	-1,94	0,055	2,22
T1 C	0,093	0,234	0,40	0,691	2,22
T1 D	0,242	0,234	1,03	0,305	2,22
T1 E	-0,137	0,234	-0,59	0,559	2,22
Treatment*Year					
K 2023	-0,171	0,148	-1,15	0,251	1,78
K 2024	0,025	0,148	0,17	0,865	1,78
T1 2023	-0,055	0,148	-0,37	0,713	1,78
T1 2024	0,006	0,148	0,04	0,968	1,78
CultivarCode*Year					
A 2023	1,035	0,234	4,42	0,000	2,22
A 2024	0,708	0,234	3,02	0,003	2,22
B 2023	-0,098	0,234	-0,42	0,677	2,22
B 2024	0,497	0,234	2,12	0,036	2,22
C 2023	-0,301	0,234	-1,28	0,202	2,22
C 2024	-0,107	0,234	-0,46	0,649	2,22
D 2023	-0,919	0,234	-3,92	0,000	2,22
D 2024	0,175	0,234	0,74	0,458	2,22
E 2023	0,230	0,234	0,98	0,329	2,22
E 2024	-0,628	0,234	-2,68	0,008	2,22

Regression Equation

Root Collar Diameter (mm) = 7,1356 - 0,575 Treatment_K + 0,033 Treatment_T1
+ 0,542 Treatment_T2 + 2,181 CultivarCode_A
+ 2,992 CultivarCode_B + 0,063 CultivarCode_C
- 0,486 CultivarCode_D - 2,128 CultivarCode_E
- 2,622 CultivarCode_F - 0,036 Year_2023 + 1,109 Year_2024
- 1,073 Year_2025 - 0,197 Treatment*CultivarCode_K A
- 0,491 Treatment*CultivarCode_K B
- 0,025 Treatment*CultivarCode_K C
+ 0,293 Treatment*CultivarCode_K D
- 0,067 Treatment*CultivarCode_K E
+ 0,487 Treatment*CultivarCode_K F
+ 0,516 Treatment*CultivarCode_T1 A
- 0,454 Treatment*CultivarCode_T1 B
+ 0,093 Treatment*CultivarCode_T1 C
+ 0,242 Treatment*CultivarCode_T1 D
- 0,137 Treatment*CultivarCode_T1 E
- 0,260 Treatment*CultivarCode_T1 F
- 0,320 Treatment*CultivarCode_T2 A
+ 0,945 Treatment*CultivarCode_T2 B
- 0,068 Treatment*CultivarCode_T2 C
- 0,535 Treatment*CultivarCode_T2 D
+ 0,204 Treatment*CultivarCode_T2 E
- 0,226 Treatment*CultivarCode_T2 F - 0,171 Treatment*Year_K 2023
+ 0,025 Treatment*Year_K 2024 + 0,146 Treatment*Year_K 2025
- 0,055 Treatment*Year_T1 2023 + 0,006 Treatment*Year_T1 2024
+ 0,049 Treatment*Year_T1 2025 + 0,225 Treatment*Year_T2 2023
- 0,031 Treatment*Year_T2 2024 - 0,194 Treatment*Year_T2 2025
+ 1,035 CultivarCode*Year_A 2023 + 0,708 CultivarCode*Year_A 2024
- 1,744 CultivarCode*Year_A 2025 - 0,098 CultivarCode*Year_B 2023
+ 0,497 CultivarCode*Year_B 2024 - 0,399 CultivarCode*Year_B 2025
- 0,301 CultivarCode*Year_C 2023 - 0,107 CultivarCode*Year_C 2024
+ 0,408 CultivarCode*Year_C 2025 - 0,919 CultivarCode*Year_D 2023
+ 0,175 CultivarCode*Year_D 2024 + 0,745 CultivarCode*Year_D 2025
+ 0,230 CultivarCode*Year_E 2023 - 0,628 CultivarCode*Year_E 2024
+ 0,398 CultivarCode*Year_E 2025 + 0,053 CultivarCode*Year_F 2023
- 0,645 CultivarCode*Year_F 2024 + 0,592 CultivarCode*Year_F 2025

Fits and Diagnostics for Unusual Observations

Obs	Root Collar Diameter (mm)	Fit	Resid	Std Resid	
12	3,230	5,072	-1,842	-2,20	R
38	13,380	10,810	2,570	3,06	R
44	9,100	10,810	-1,710	-2,04	R
87	9,600	7,910	1,690	2,02	R
92	14,500	11,689	2,811	3,35	R
98	9,500	11,689	-2,189	-2,61	R
146	9,400	7,098	2,302	2,75	R
154	6,000	7,735	-1,735	-2,07	R
155	6,100	8,283	-2,183	-2,60	R
156	8,200	9,948	-1,748	-2,09	R
161	10,100	8,283	1,817	2,17	R

R Large residual

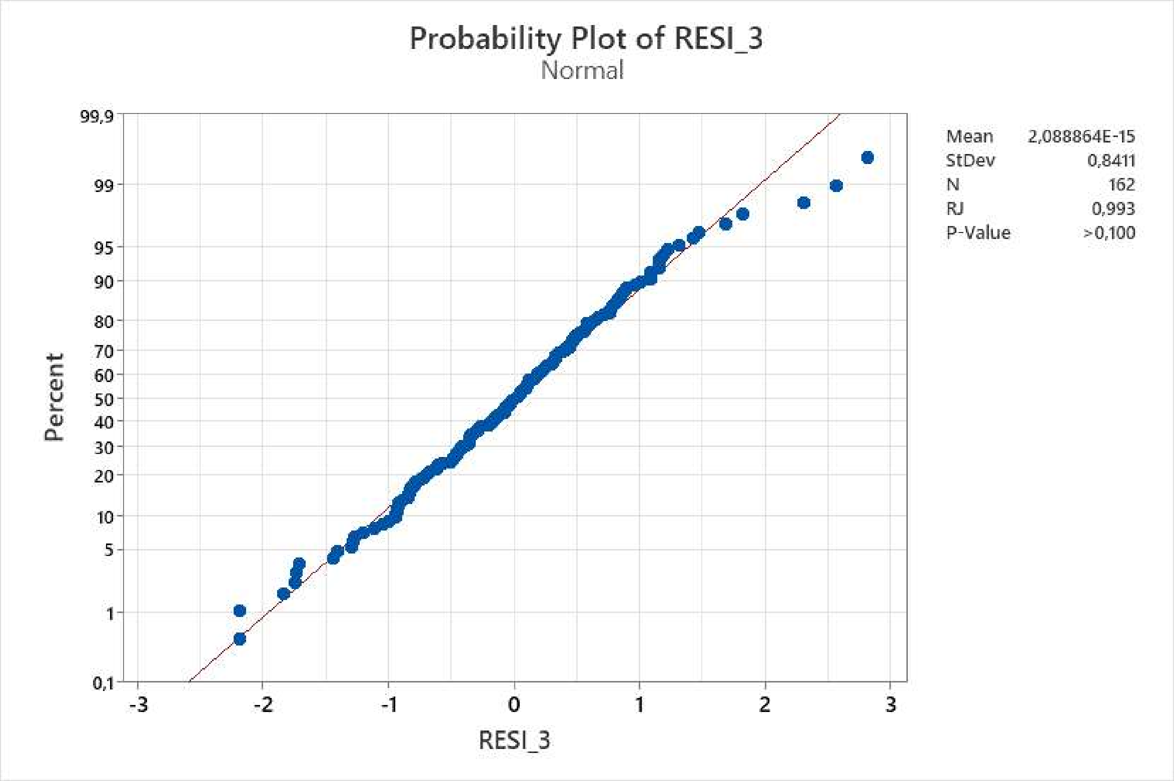

Method

Null hypothesis	All variances are equal
Alternative hypothesis	At least one variance is different
Significance level	α = 0,05

95% Bonferroni Confidence Intervals for Standard Deviations

Treatment	N	StDev	CI
K	54	2,20967	(1,84068; 2,77568)
T1	54	2,62459	(2,08288; 3,46060)

T2	54	2,75422	(2,28449; 3,47457)

Individual confidence level = 98,3333%

Tests

Method	Test Statistic	P-Value
Multiple comparisons	—	0,168
Levene	0,89	0,414

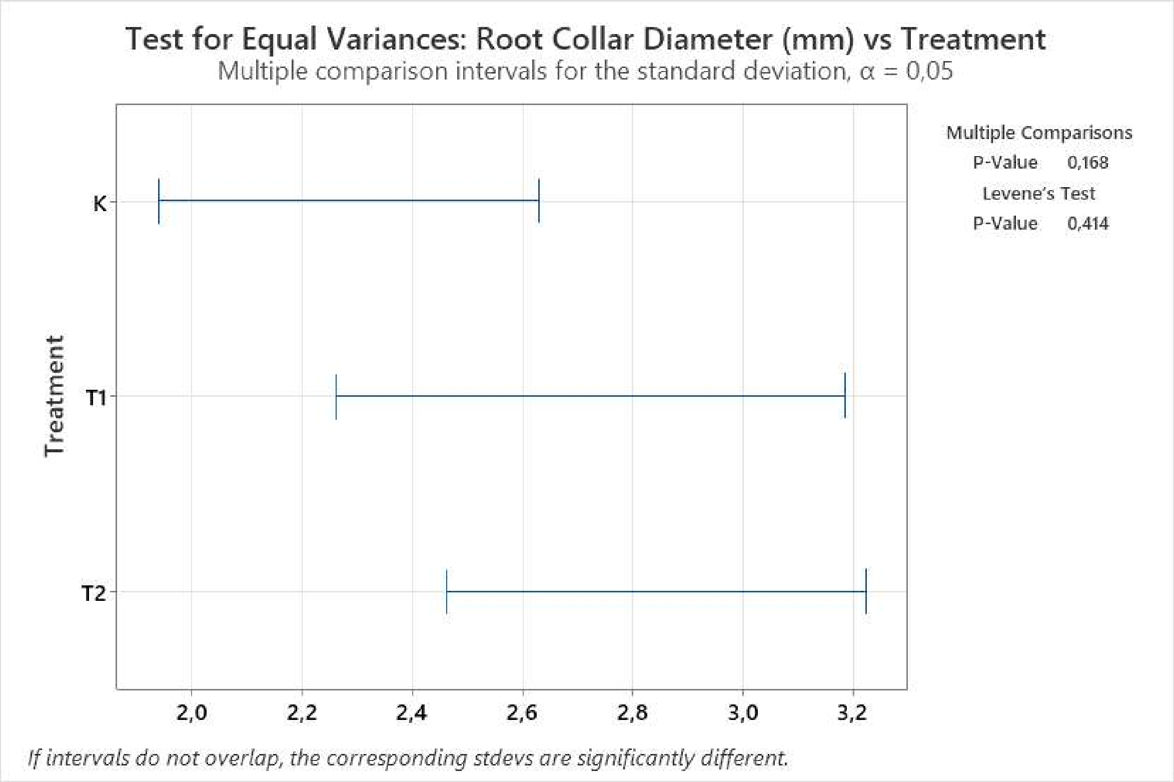

Supplement: Supplementary file 1 [file plants-15-00803-s001.zip › S4 - Root Collar Diameter (mm) - GLM+Probability Plot+Test for Equal Variances.pdf]
