# Supplementary material for: Microalgae-Based Biostimulants Improve Biomass Production and Root-Linked Performance Stability in Pelargonium: A Three-Year Greenhouse Study
Source: Plants (Basel). 2026 Mar 5;15(5):803. doi: 10.3390/plants15050803 (PMC12986712; doi:10.3390/plants15050803)
Supplement: Supplementary file 1 [file plants-15-00803-s001.zip › S6 - Descriptive Statistics collection.pdf]

Descriptive Statistics Summary

Statistics

Variable	Treatment	N	Mean	SE Mean	StDev	Minimum	Maximum
Dry Plant Mass (g)	K	54	3,52778	0,135991	0,999324	1,8	6,5
	T1	54	4,20630	0,155241	1,14078	2	7,2
	T2	54	4,69537	0,152365	1,11965	2,2	7,6

Dry plant mass (g) *(Formula: Δ = Mean (Tx) – Mean(K); Δ% = 100×Δ/Mean(K)*

- K (Control): 3.52778
- T1 (MACC-612, Nostoc piscinale): 4.20630 → Δ = +0.67852 g, Δ% = **+19.2%**
- T2 (MACC-922, Chlorella vulgaris): 4.69537 → Δ = +1.16759 g, Δ% = **+33.1%**

Statistics

Variable	Treatment	N	Mean	SE Mean	StDev	Minimum	Maximum
Root Mass (g)	K	54	1,50815	0,0882694	0,648645	0,5	3,31
	T1	54	1,84463	0,108724	0,798951	0,4	3,8
	T2	54	1,87130	0,105491	0,775196	0,49	3,91

Root mass (g) *(Formula: Δ = Mean (Tx) – Mean(K); Δ% = 100×Δ/Mean(K)*

- K (Control): 1.50815
- T1 (MACC-612, Nostoc piscinale): 1.84463 → Δ = +0.33648 g, Δ% = **+22.3%**
- T2 (MACC-922, Chlorella vulgaris): 1.87130 → Δ = +0.36315 g, Δ% = **+24.1%**

Statistics

Variable	Treatment	N	Mean	SE Mean	StDev	Minimum	Maximum
Root Collar Diameter (mm)	K	54	6,56037	0,300698	2,20967	2,8	11,5
	T1	54	7,16852	0,357161	2,62459	3,31	14,5
	T2	54	7,67796	0,374802	2,75422	3,23	13,5

Root collar diameter (mm) *(Formula: Δ = Mean (Tx) – Mean(K); Δ% = 100×Δ/Mean(K)*

- K (Control): 6.56037
- T1 (MACC-612, Nostoc piscinale): 7.16852 → Δ = +0.60815 mm, Δ% = **+9.3%**
- T2 (MACC-922, Chlorella vulgaris): 7.67796 → Δ = +1.11759 mm, Δ% = **+17.0%**

Statistics

Variable	Treatment	N	Mean	SE Mean	StDev	Minimum	Maximum
Plant Height (cm)	K	54	22,9130	0,721580	5,30251	10,5	33
	T1	54	25,0222	0,490209	3,60229	17,5	33,5
	T2	54	24,7426	0,776609	5,70688	12,5	38

Plant height (cm) *(Formula: Δ = Mean (Tx) – Mean(K); Δ% = 100×Δ/Mean(K)*

- K (Control): 22.9130
- T1 (MACC-612, Nostoc piscinale): 25.0222 → Δ = +2.1092 cm, Δ% = **+9.2%**
- T2 (MACC-922, Chlorella vulgaris): 24.7426 → Δ = +1.8296 cm, Δ% = **+8.0 %**
