# Supplementary material for: Microalgae-Based Biostimulants Improve Biomass Production and Root-Linked Performance Stability in Pelargonium: A Three-Year Greenhouse Study
Source: Plants (Basel). 2026 Mar 5;15(5):803. doi: 10.3390/plants15050803 (PMC12986712; doi:10.3390/plants15050803)
Supplement: Supplementary file 1 [file plants-15-00803-s001.zip › Supplementary Materials Legend.pdf]

Supplementary Materials

Supplementary Materials S1: Dry Plant Mass (g)—GLM + Probability Plot + Test for Equal Variances (PDF).
Supplementary Materials S2: Root Mass (g)—GLM + Probability Plot + Test for Equal Variances (PDF).
Supplementary Materials S3: Plant Height (cm)—GLM + Probability Plot + Test for Equal Variances (PDF).
Supplementary Materials S4: Root Collar Diameter (mm)—GLM + Probability Plot + Test for Equal Variances (PDF).
Supplementary Materials S5: Environmental data (XLSX).
Supplementary Materials S6: Descriptive Statistics collection (PDF).
Supplementary Materials S7: Base data for End-Point Measurements (XLSX).
Supplementary Materials S8: Cultivar-level effect size (XLSX).
